# Modulating the immune response to SARS-CoV-2 by different nanocarriers delivering an mRNA expressing trimeric RBD of the spike protein: COVARNA Consortium

**DOI:** 10.1038/s41541-024-00838-8

**Published:** 2024-03-06

**Authors:** Laura Marcos-Villar, Beatriz Perdiguero, Shubaash Anthiya, Mireya L. Borrajo, Gustavo Lou, Lorenzo Franceschini, Ignasi Esteban, Pedro J. Sánchez-Cordón, Carmen Zamora, Carlos Óscar S. Sorzano, Luis Jordá, Laia Codó, Josep L. Gelpí, Marta Sisteré-Oró, Andreas Meyerhans, Kris Thielemans, Francisco Martínez-Jiménez, Núria López-Bigas, Felipe García, María J. Alonso, Montserrat Plana, Mariano Esteban, Carmen Elena Gómez

**Affiliations:** 1 Department of Molecular and Cellular Biology, Centro Nacional de Biotecnología, Consejo Superior de Investigaciones Científicas, Madrid, Spain; 2 Centro de Investigación Biomédica en Red de Enfermedades Infecciosas (CIBERINFEC), Instituto de Salud Carlos III (ISCIII), Madrid, Spain; 3 Center for Research in Molecular Medicine and Chronic Diseases (CiMUS), Campus Vida, Universidade de Santiago de Compostela, Santiago de Compostela, Spain; 4 Laboratory for Molecular and Cellular Therapy, Department of Biomedical Sciences, Vrije Universiteit Brussel, Brussels, Belgium; 5 AIDS Research Group, Institut d’Investigacions Biomèdiques August Pi i Sunyer (IDIBAPS), Hospital Clinic, University of Barcelona, Barcelona, Spain; 6 Veterinary Pathology Department, Centro de Investigación en Sanidad Animal, Instituto Nacional de Investigación y Tecnología Agraria y Alimentaria, Consejo Superior de Investigaciones Científicas, Madrid, Spain; 7 Biocomputing Unit and Computational Genomics, Centro Nacional de Biotecnología, Consejo Superior de Investigaciones Científicas, Madrid, Spain; 8 Barcelona Supercomputing Center (BSC), Barcelona, Spain; 9 Department of Biochemistry and Molecular Biomedicine, University of Barcelona, Barcelona, Spain; 10 Infection Biology Laboratory, Department of Medicine and Life Sciences, University Pompeu Fabra, Barcelona, Spain; 11 Institució Catalana de Recerca i Estudis Avançats (ICREA), Barcelona, Spain; 12 Institute for Research in Biomedicine (IRB Barcelona), The Barcelona Institute of Science and Technology, Barcelona, Spain; 13 Centro de Investigación Biomédica en Red en Cáncer (CIBERONC), Instituto de Salud Carlos III, Madrid, Spain; 14 Infectious Diseases Department, Hospital Clínic, University of Barcelona, Barcelona, Spain

**Keywords:** Viral infection, RNA vaccines

## Abstract

Vaccines based on mRNA technology have revolutionized the field. In fact, lipid nanoparticles (LNP) formulated with mRNA are the preferential vaccine platform used in the fight against SARS-CoV-2 infection, with wider application against other diseases. The high demand and property right protection of the most potent cationic/ionizable lipids used for LNP formulation of COVID-19 mRNA vaccines have promoted the design of alternative nanocarriers for nucleic acid delivery. In this study we have evaluated the immunogenicity and efficacy of different rationally designed lipid and polymeric-based nanoparticle prototypes against SARS-CoV-2 infection. An mRNA coding for a trimeric soluble form of the receptor binding domain (RBD) of the spike (S) protein from SARS-CoV-2 was encapsulated using different components to form nanoemulsions (NE), nanocapsules (NC) and lipid nanoparticles (LNP). The toxicity and biological activity of these prototypes were evaluated in cultured cells after transfection and in mice following homologous prime/boost immunization. Our findings reveal good levels of RBD protein expression with most of the formulations. In C57BL/6 mice immunized intramuscularly with two doses of formulated RBD-mRNA, the modified lipid nanoparticle (mLNP) and the classical lipid nanoparticle (LNP-1) were the most effective delivery nanocarriers at inducing binding and neutralizing antibodies against SARS-CoV-2. Both prototypes fully protected susceptible K18-hACE2 transgenic mice from morbidity and mortality following a SARS-CoV-2 challenge. These results highlight that modulation of mRNAs immunogenicity can be achieved by using alternative nanocarriers and support further assessment of mLNP and LNP-1 prototypes as delivery vehicles for mRNA vaccines.

## Introduction

The ongoing global coronavirus disease 2019 (COVID-19) pandemic caused by the severe acute respiratory syndrome coronavirus-2 (SARS-CoV-2) has caused more than 774 million cases and 7 million confirmed deaths on 11 February 2024 (https://covid19.who.int), being one of the deadliest virus diseases in history. Fortunately, the extraordinarily rapid development, manufacturing and worldwide administration of COVID‑19 vaccines have significantly impacted the prevention and transmission of the disease. In fact, authorized vaccines still offer good protection against severe disease, hospitalization and death caused by the appearance of new SARS-CoV-2 variants^1^. The first two vaccines that received FDA and EMA approval against COVID-19 were developed by Pfizer-BioNTech (BNT162b2) and Moderna (mRNA-1273) companies using an mRNA technology platform not previously authorized for human use. BNT162b2 and mRNA-1273 are lipid nanoparticle (LNP)-formulated, nucleoside-modified mRNA vaccines that encode a prefusion stabilized, membrane-anchored full-length spike (S) protein from SARS-CoV-2. They have been reported to be safe, immunogenic and remarkably effective against severe disease, hospitalization and death across age groups and in diverse populations but exhibited moderate efficacy against symptomatic SARS-CoV-2 infection^2^.

The success of the COVID-19 mRNA vaccines has provided remarkable proof of concept of the potential of this platform to rapidly respond to public health emergencies of infectious diseases^3^. However, mRNA technology still needs to improve some critical points related with mRNA stability, intracellular delivery, immunogenicity, efficiency of in vivo protein expression and scale up production.

The delivery system plays a key role in protecting mRNA structure, promoting cellular internalization through endocytosis, and ensuring the efficacy and safety of mRNA-based vaccines. LNPs are, clinically, the most advanced mRNA carriers^4^. The composition of an LNP formulation can define the cell specificity of delivery, significantly affect the intracellular delivery efficiency and modulate immunogenicity, being the cationic/ionizable lipid a critical component to condense the mRNA molecules and facilitate their endosomal escape by disrupting the cell membrane^5^.

Considering the importance of the formulation of the mRNA molecules in appropriate nanocarriers, in this study we have evaluated the immunogenicity and efficacy of different rationally designed nanocarrier prototypes against SARS-CoV-2 infection. An mRNA encoding a trimeric soluble form of the receptor binding domain (RBD) of the S protein from SARS-CoV-2 was encapsulated using different components to form nanoemulsions (NE), nanocapsules (NC) and LNP^6^. RBD protein was detected in vitro in cells transfected with most of the formulations, whereas in C57BL/6 mice only the RBD-mRNA delivered by two LNP prototypes (mLNP and LNP-1) elicited SARS-CoV-2-specific binding and neutralizing antibodies against the original ancestral SARS-CoV-2 strain and against different variants of concern (VoCs). These two nanocarriers fully protected susceptible transgenic K18-hACE2 mice from morbidity and mortality following a SARS-CoV-2 challenge. These results point out that a modulation of the immunogenicity elicited by mRNAs can be fulfilled by using alternative nanocarriers and reinforce further evaluation of mLNP and LNP-1 prototypes as delivery vehicles for mRNA vaccines.

Currently, researchers in the drug delivery field are actively working on the optimization of LNPs and the design of alternative nanocarriers^7–9^. Our approach in this line has relied on the modification of the composition of original standard LNPs as well as on the development of distinct nanocarriers, such as NEs and NCs, that are composed of regulatory acceptable biomaterials and that, due to their simplicity, could be easily translated to a global context.

## Results

### Validation of three forms of mRNA expressing RBD of SARS-CoV-2

We generated three forms of mRNA encoding the RBD of SARS-CoV-2 virus (Supplementary Fig. 1A) with the aim to define the best-in-class when transfected in cultured cells. The kinetics of expression of RBD protein from 293T cells transfected with the three forms of lipofectamine-treated RBDepi-mRNA, RBD-mRNA or modified RBD-mRNA* was determined by flow cytometry and western-blotting analyses using a rabbit polyclonal anti-SARS-CoV-2 spike/RBD antibody. As shown in Supplementary Fig. 1B, RBD expression was detected within cells as early as 3 h post-transfection for all mRNAs assayed, and their expression increased with time, peaking at 6 h. The highest level of RBD expression was detected in cells transfected with unmodified RBD-mRNA that encodes the SARS-CoV-2 S protein RBD fused to the T4 fibritin trimerization foldon. The lower level of RBD expression observed in RBD-mRNA*-transfected cells indicates that the modification of the mRNA using 1-methyl-3′-pseudouridylyl instead of UTP does not improve the in vitro translation and expression of the RBD protein in this system.

By western-blotting analysis, RBD expression was detected in the cellular pellets 3 h post-transfection, peaking at 24 h (Supplementary Fig. 1C, left panel). In the supernatants of transfected cells, the three forms of RBD protein were clearly detected at 6 h, with unmodified RBD-mRNA showing the highest levels of RBD expression at 6 and 24 h post-transfection (Supplementary Fig. 1C, right panel). These results indicate that SARS-CoV-2 RBD from S protein is well expressed in cultured cells from three distinct forms of mRNAs, with the higher expression being observed with unmodified mRNA.

### Formulated RBD-mRNA was efficiently delivered and translated by different nanocarriers

**Fig. 1:**
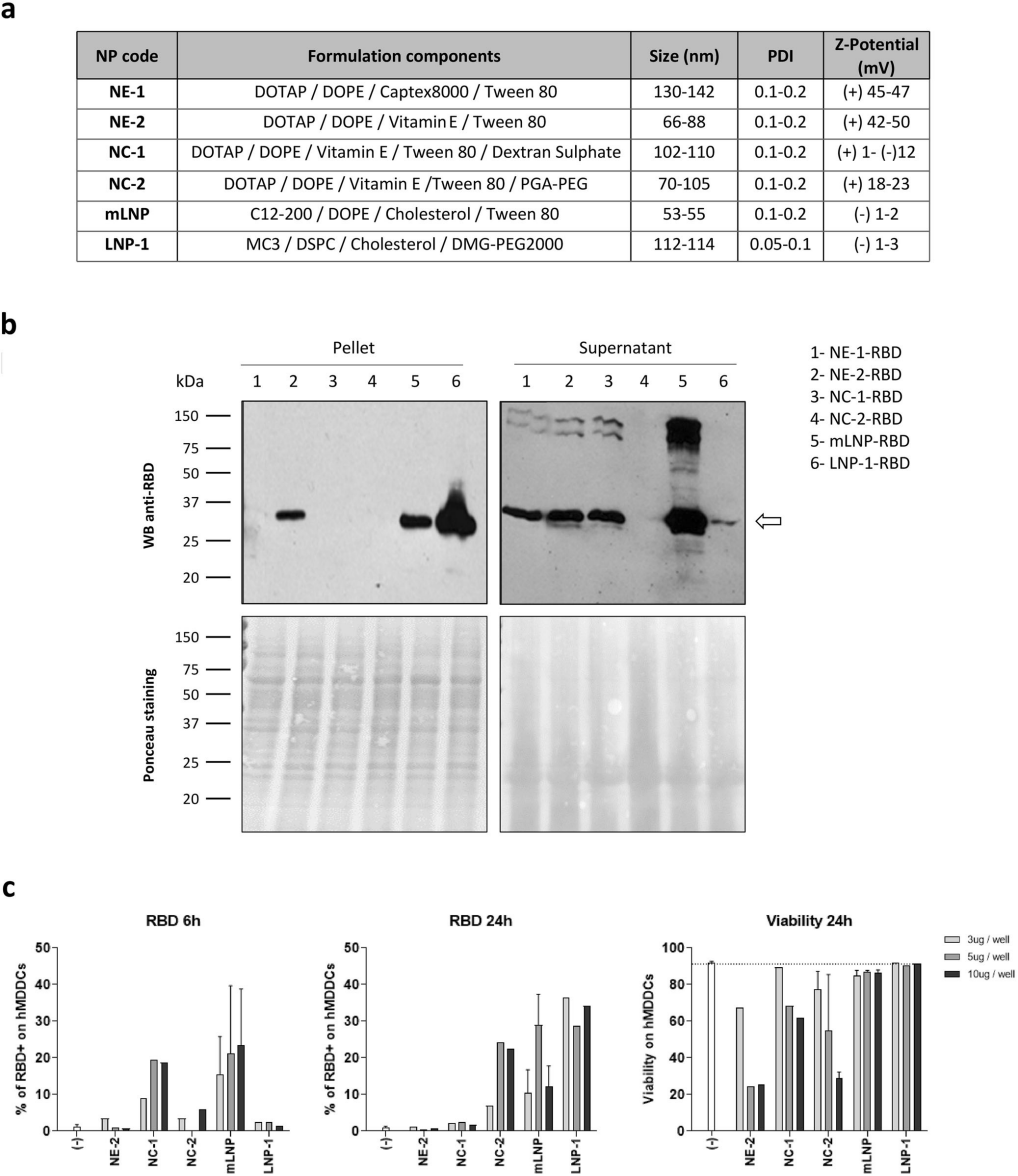
Expression of SARS-CoV-2 RBD protein in cells transfected with formulated RBD-mRNAs. **a** Description of the different nanocarriers used for the encapsulation of RBD-mRNA. **b** Detection of RBD expression in cellular pellets and supernatants from 293T cells transfected with the different formulated RBD-mRNAs for 6 h by western-blotting analysis using a rabbit polyclonal anti-SARS-CoV-2 spike/RBD antibody (upper panels). Ponceau staining (lower panels) was used as loading control. All blots derive from the same experiment and were processed in parallel. **c** RBD expression and viability of human monocyte-derived dendritic cells (hMDDCs) from a healthy donor at 6 and 24 h after transfection with the different nanocarriers containing the RBD-mRNA. Mean with standard error of the mean (SEM) is represented.

Since the unmodified RBD-mRNA showed the highest RBD expression levels in transfected cells, we decided to use it as a model mRNA candidate to be formulated in different nanocarriers. For this, purified RBD-mRNA was encapsulated in 2 nanoemulsions (NE-1 and NE-2), 2 nanocapsules (NC-1 and NC-2) and 2 lipid nanoparticles (mLNP and LNP-1) using the solvent-displacement technique, applied by either hand-mixing or microfluidics, as described in Materials and Methods. The delivery and expression of RBD-mRNA from the different nanocarriers described in Fig. 1a was analyzed by western-blotting in the cellular pellets and supernatants from 293T cells transfected for 6 h with the different formulations. As shown in Fig. 1b, the RBD protein (28.5 kDa) was mostly detected in the supernatant of transfected cells, although for the LNP-1-RBD prototype the highest expression level was detected in the cellular pellet. This change might reflect a differential kinetics of formulated RBD-mRNA delivery into the cell, which can in turn depend on several factors including lipid composition, ultrastructure of the nanocarrier and mRNA packaging. In cells transfected with the nanocapsule NC-2-RBD, RBD protein could not be detected in the cellular pellet nor in the supernatant of transfected cells.

### Differential transfection efficiency and toxicity of different nanocarriers in hMDDCs

RBD mRNA formulations were also tested in hMDDCs to assess the activity on primary antigen-presenting cells (APCs). hMDDCs were obtained from isolated blood monocytes after 6 days of in vitro culture in the presence of IL-4 and GM-CSF. After, hMDDCs were harvested and transfected with the RBD formulations, assessing the intracellular expression of RBD protein and the in vitro toxicity by flow cytometry at 6 and 24 h post-transfection (Fig. 1c). Expression of RBD on hMDDCs was detectable with all tested formulations except for NE-2-RBD. The NC-2-RBD, mLNP-RBD and LNP-1-RBD induced high expression levels at 24 h. Of notice, the experiment showed important differences in the kinetics of RBD expression between formulations. For example, with NC-1-RBD, the RBD protein was only detectable at 6 h, while with NC-2-RBD and LNP-1-RBD, RBD was observed at 24 h but not at 6 h. On the other hand, with mLNP-RBD, RBD was detected at both 6 and 24 h. These differences in the mRNA expression kinetics could be related to differences in lipid composition, the cationic lipid used (DOTAP or C12-200), pKas, PEG density, ultrastructure of the nanoparticles, membrane fluidity, interaction between lipids-mRNA or mRNA packaging, among others^10^. Regarding toxicity, mLNP-RBD and LNP-1-RBD were the less toxic formulations, showing no signs of increased cell mortality even when cells were transfected with the highest concentration (10 μg/well). The rest of the formulations exhibited some degree of toxicity, with NE-2-RBD being the most toxic, possibly due to its highly positive charge and lipid composition. Therefore, mLNP and LNP-1 are the nanocarriers that combine the highest capacity to induce RBD expression with the lowest toxicity profile.

### LNPs induced RBD-specific IgG and SARS-CoV-2 neutralizing antibodies after homologous prime/boost vaccination in C57BL/6 mice

**Fig. 2:**
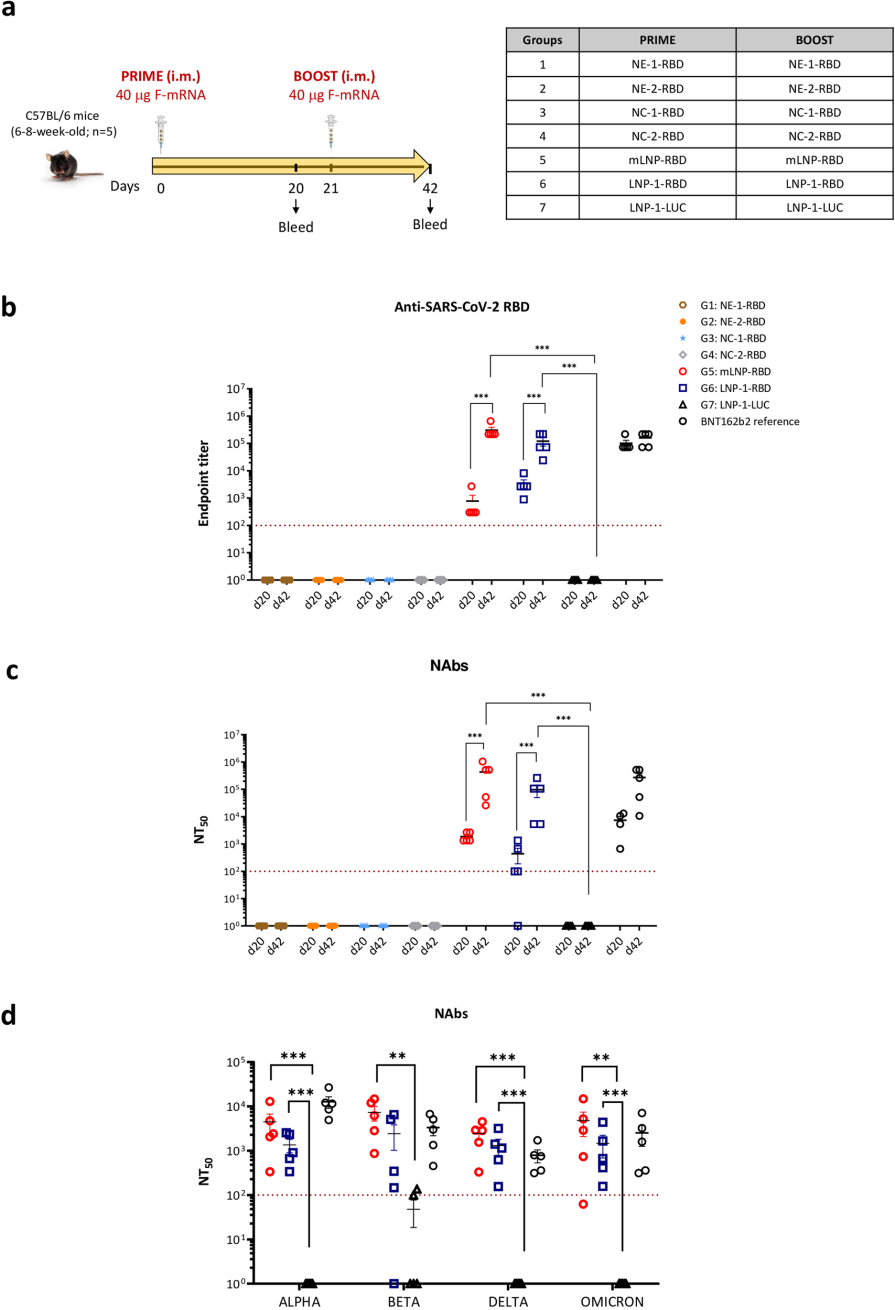
Humoral immune responses induced in C57BL/6 mice by different nanocarriers containing RBD-mRNA. **a** Immunization schedule. Female C57BL/6 mice (*n* = 5) were immunized with two doses of 40 µg of the different formulations containing RBD-mRNA by intramuscular (i.m.) route as indicated. **b** SARS-CoV-2 RBD-specific IgG binding antibodies. Anti-RBD IgG titers were determined in individual sera obtained at 20 days post-prime (d20) or 21 days post-boost (d42) by ELISA. An unpaired nonparametric Mann–Whitney test of transformed data was used. ***p < 0.001. **c** SARS-CoV-2 neutralizing antibody responses. NT_50_ titers were determined in individual sera harvested at d20 and d42 using a live virus microneutralization assay (MAD6 strain, containing D614G mutation). An ordinary one-way ANOVA of transformed data followed by Tukey’s multiple comparison test was performed. ***p < 0.001. **d** Neutralizing antibody responses induced against SARS-CoV-2 variants. NT_50_ titers were evaluated in individual serum samples harvested at d42 by a live virus microneutralization assay using the SARS-CoV-2 Alpha (B.1.1.7), Beta (B.1.351), Delta (B.1.617.2) and Omicron (B.1.1.529) variants. An ordinary one-way ANOVA of transformed data followed by Tukey’s multiple comparison test was performed. **p < 0.01; ***p < 0.001. Serum samples from mice similarly vaccinated with two doses of 5 μg of BNT162b2 vaccine (mRNA vaccine from Pfizer-BioNTech) were used as a reference value (BNT162b2 reference). Red dashed line represents the lower limit of detection of the assay. Mean with standard error of the mean (SEM) is represented.

Once demonstrated that the different nanocarriers delivered the RBD-mRNA into the cells with production of the RBD protein, we evaluated the potential of these formulations to induce SARS-CoV-2-specific humoral immune responses in the mouse model. For this, groups of C57BL/6 mice were immunized with two intramuscular doses of the corresponding prototype at days 0 and 21. The schedule and immunization groups are depicted in Fig. 2a. At 20 days post-prime (d20) and 21 days post-boost (d42) mice were bled, serum collected, and the levels of SARS-CoV-2 RBD-specific IgG binding and neutralizing antibodies were determined by ELISA and microneutralization assay, respectively.

As shown in Fig. 2b, c, most of the formulations, except mLNP-RBD and LNP-1-RBD, failed to induce a SARS-CoV-2-specific humoral immune response despite having demonstrated in cultured cells their potential to allow the delivery and translation of RBD-mRNA. In contrast, both LNP prototypes efficiently enhanced both SARS-CoV-2 RBD-specific IgG binding antibodies and SARS-CoV-2 (MAD6) neutralizing antibodies (NAbs). After the first dose all animals seroconverted, with SARS-CoV-2 RBD-specific binding antibody titers ranging from 300 to 2,700 for mLNP-RBD and from 900 to 8,100 for LNP-1-RBD. These values were significantly enhanced after the second homologous boost for both groups, reaching anti-RBD IgG titers from 218,700 to 656,100 for mLNP-RBD and from 24,300 to 218,700 for LNP-1-RBD (Fig. 2b). Similarly, after one dose animals in both mLNP-RBD and LNP-1-RBD groups elicited anti-SARS-CoV-2 NAbs with NT_50_ titers ranging between 1300 and 2600 for mLNP-RBD and from 100 to 1300 for LNP-1-RBD. The second dose significantly increased these levels in all animals reaching NT_50_ titers from 26,000 to 1,040,000 for mLNP-RBD and from 5300 to 260,000 for LNP-1-RBD (Fig. 2c). The neutralizing capacity of the sera from mLNP-RBD- and LNP-1-RBD-immunized animals against different SARS-CoV-2 VoCs was also evaluated after the boost (d42). As shown in Fig. 2d, high neutralizing antibody titers were detected in both groups against Alpha (B.1.1.7), Beta (B.1.351), Delta (B.167.2) and Omicron (B.1.1.529) SARS-CoV-2 variants, with a trend to higher neutralizing antibody levels in the group of mice immunized with mLNP-RBD. The levels of SARS-CoV-2-specific binding and neutralizing antibodies detected in both mLNP-RBD and LNP-1-RBD immunization groups are comparable to those detected in mice similarly vaccinated with two doses of 5 μg of BNT162b2 vaccine.

In summary, homologous prime/boost combination of both LNP-based formulated RBD-mRNAs induced high levels of humoral responses (binding and neutralizing antibodies against MAD6 and VoCs) in vaccinated C57BL/6 mice.

### LNP-RBD formulations induced high levels of SARS-CoV-2 S- and RBD-specific IgGs and neutralizing antibodies against SARS-CoV-2 MAD6 strain and different SARS-CoV-2 VoCs in transgenic K18-hACE2 mice before virus challenge

**Fig. 3:**
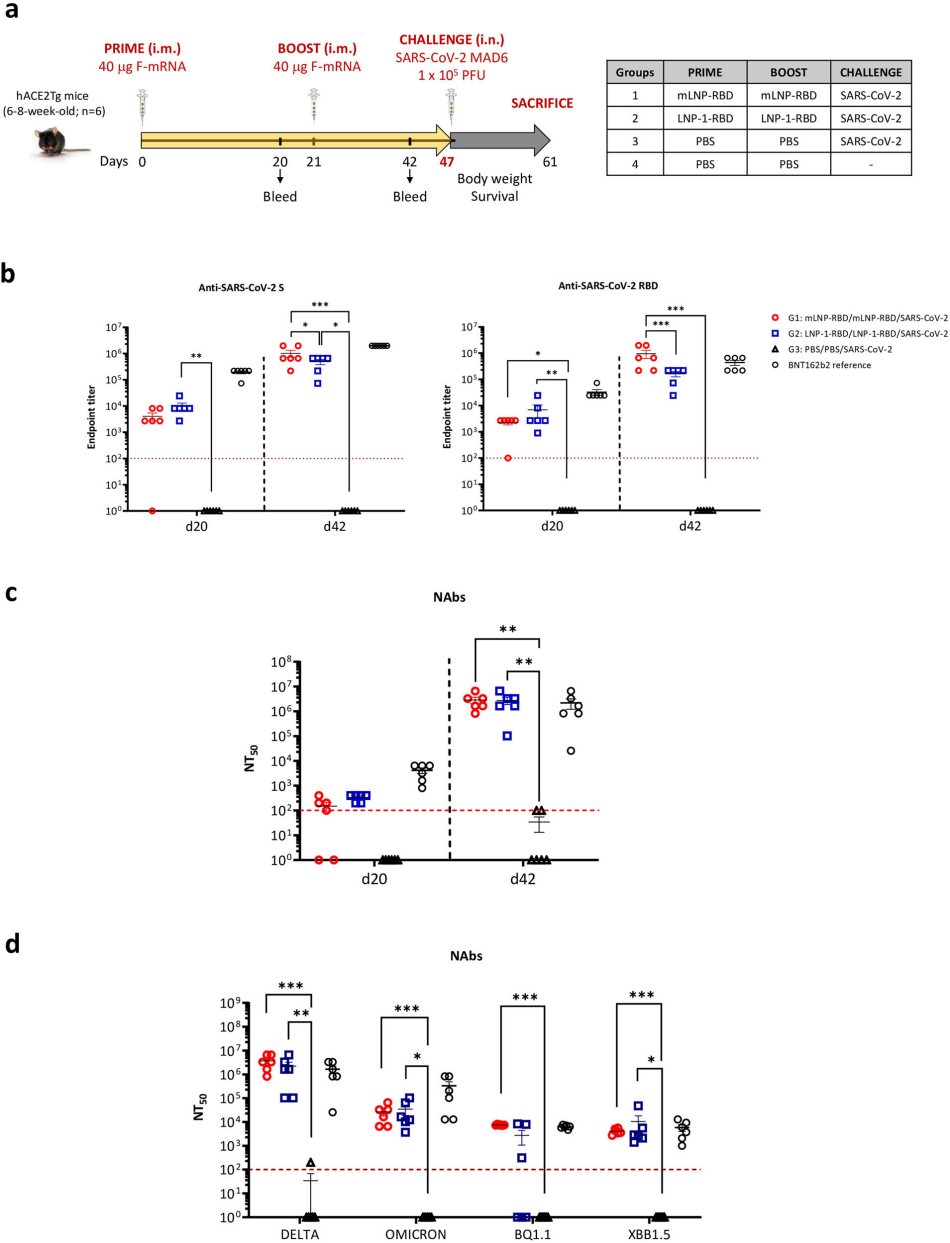
SARS-CoV-2-specific humoral responses elicited in transgenic K18-hACE2 mice by mLNP-RBD or LNP-1-RBD formulations before virus challenge. **a** Immunization schedule. K18-hACE2 transgenic mice (*n* = 6) were immunized with two doses of 40 µg of mLNP-RBD or LNP-1-RBD formulations by i.m. route as indicated. At day 47 mice were challenged intranasally (i.n.) with 1 × 10^5^ PFU of SARS-CoV-2 (MAD6 isolate, containing D614G mutation). **b** SARS-CoV-2 S- and RBD-specific IgG binding antibodies. Anti-S and anti-RBD IgG titers were determined in individual sera obtained at 20 days post-prime (d20) or 21 days post-boost (d42) by ELISA. An unpaired nonparametric Mann-Whitney test of transformed data was used. *p < 0.05; **p < 0.005; ***p < 0.001. **c** SARS-CoV-2 neutralizing antibody responses. NT_50_ titers were determined in individual sera harvested at d20 and d42 using a live virus microneutralization assay (MAD6 strain, containing D614G mutation). An ordinary one-way ANOVA of transformed data followed by Tukey’s multiple comparison test was performed. **p < 0.005. **d** Neutralizing antibody responses induced against SARS-CoV-2 variants. NT_50_ titers were evaluated in individual sera collected at d42 by a live virus microneutralization assay using the SARS-CoV-2 Delta (B.1.617.2), Omicron (B.1.1.529), BQ1.1 and XBB1.5 variants. An ordinary one-way ANOVA of transformed data followed by Tukey’s multiple comparison test was performed. *p < 0.05; **p < 0.005; ***p < 0.001. Serum samples from mice similarly vaccinated with two doses of 5 μg of BNT162b2 vaccine (mRNA vaccine from Pfizer-BioNTech) were used as a reference value (BNT162b2 reference). Red dashed line represents the lower limit of detection of the assay. Mean with standard error of the mean (SEM) is represented.

To further characterize the efficacy of mLNP-RBD and LNP-1-RBD formulations against SARS-CoV-2 infection we performed a homologous prime/boost inoculation of susceptible transgenic K18-hACE2 mice according to the schedule and immunization groups indicated in Fig. 3a. We evaluated the humoral responses (binding antibodies against SARS-CoV-2 S and RBD proteins and NAbs against SARS-CoV-2 virus) induced by both prototypes at 20 days post-prime (d20) and 21 days post-boost (d42).

As shown in Fig. 3b, high titers of SARS-CoV-2 S- and RBD-specific IgG binding antibodies were elicited in mice immunized with mLNP-RBD or LNP-1-RBD after the first dose (d20). These levels were significantly enhanced after the boost (d42) in both groups (Fig. 3b). Similar behavior was observed for the SARS-CoV-2-specific NAbs against MAD6 strain of SARS-CoV-2 (Fig. 3c). The neutralizing capacity of the sera from immunized animals against different SARS-CoV-2 VoCs was also assessed after the boost (d42). Remarkably, both groups exhibited high neutralizing antibody titers against Delta SARS-CoV-2 variant, while showing comparatively lower levels of neutralization against Omicron, BQ1.1 and XBB1.5 virus strains (Fig. 3d). Again, the levels of SARS-CoV-2-specific binding and neutralizing antibodies detected in both LNP-based RBD immunization groups mirror those observed in mice similarly vaccinated with two doses of 5 μg of BNT162b2 vaccine.

In summary, homologous prime/boost combination of both LNP-based RBD-mRNAs induced high levels of humoral responses (binding and neutralizing antibodies against VoCs) in vaccinated K18-hACE2 transgenic mice before the virus challenge.

### Homologous prime/boost administration of LNPs fully protects transgenic K18-hACE2 mice from morbidity and mortality against SARS-CoV-2 infection

Next, we tested the capacity of the different RBD-mRNA LNP formulations to protect mice against SARS-CoV-2 infection. For this, we challenged intranasally the vaccinated K18-hACE2 animals with 1 × 10^5^ PFU of SARS-CoV-2 (MAD6) virus as illustrated in Fig. 3a. Mice were daily monitored for body weight and survival for 14 days. Animals that lost more than 25% of the initial weight and those that survived until the end of the experiment were sacrificed and lungs and serum samples were harvested.

**Fig. 4:**
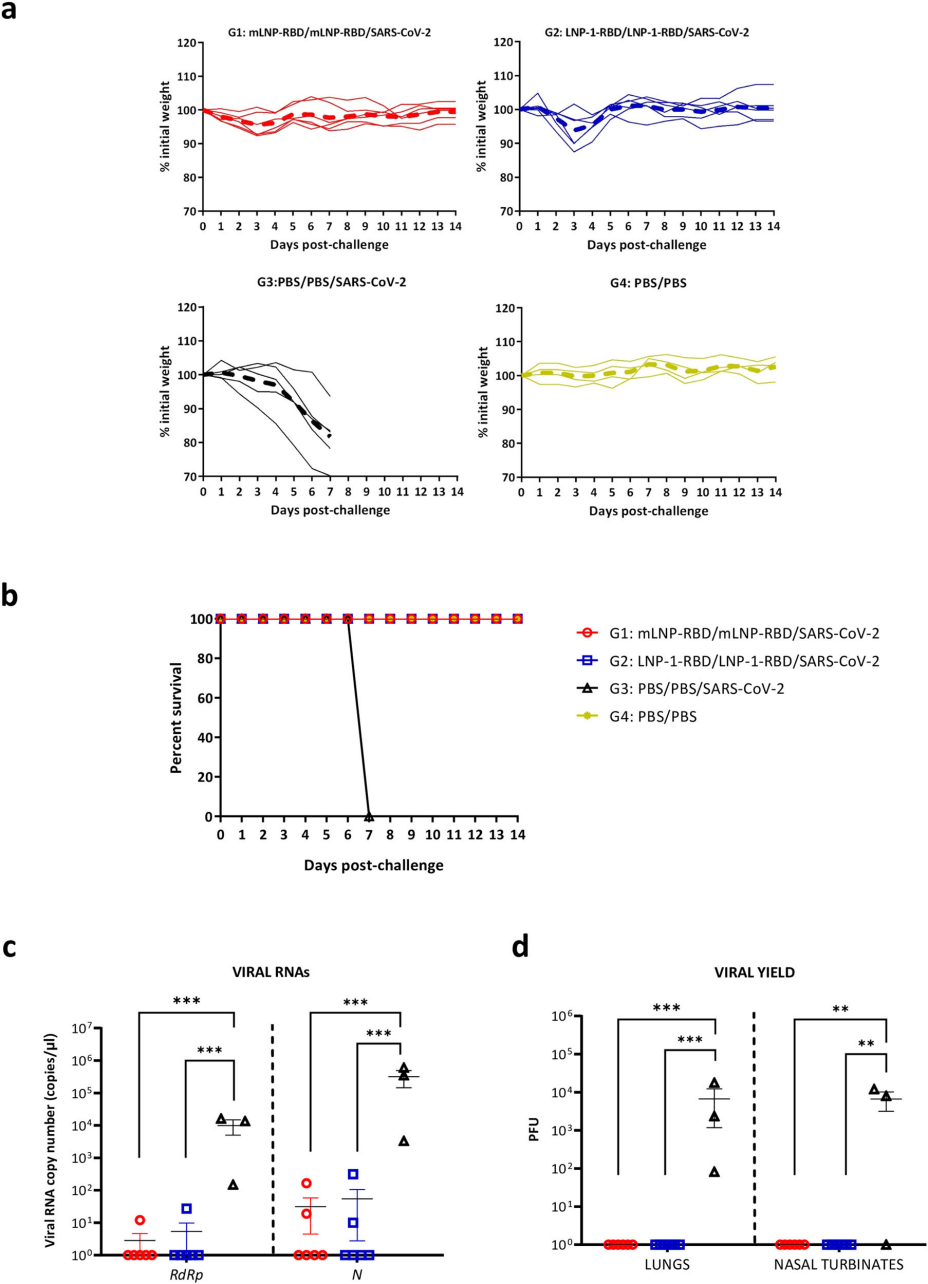
Homologous prime/boost administration of mLNP-RBD or LNP-1-RBD fully protects transgenic K18-hACE2 mice from morbidity and mortality against SARS-CoV-2 infection. Individual mice were daily monitored for changes of body weight (**a**) and mortality (**b**) for 14 days. Mice that lost more than 25% of the initial body weight were sacrificed. **c** Genomic (*RdRp*) and subgenomic (*N*) SARS-CoV-2 RNAs detected by RT-qPCR in lungs from individual mice at 14 days (groups 1 and 2) or 7 days (group 3) after SARS-CoV-2 challenge. Mean RNA copy numbers (copies/μl) with standard error of the mean (SEM) from duplicates of each lung sample is represented. Relative values are referred to uninfected mice (group 4). An ordinary one-way ANOVA of transformed data followed by Tukey’s multiple comparison test was performed. ***p < 0.001. **d** SARS-CoV-2 infectious virus in lung or nasal turbinates. Mean PFU (PFU/gram of lung tissue or PFU/mL of nasal turbinates) with SEM from triplicates of each sample is represented. An ordinary one-way ANOVA of transformed data followed by Tukey’s multiple comparison test was performed. **p < 0.005; ***p < 0.001.

As observed in Fig. 4a, b, mice vaccinated with mLNP-RBD or LNP-1-RBD did not lose body weight and survived until the end of the efficacy study similarly to the PBS-treated non-challenged mice. However, all PBS-challenged mice lost body weight progressively and had to be sacrificed at 7 days post-challenge. We also determined the presence of SARS-CoV-2 genomic *RdRp* and subgenomic *N* RNAs in individual lung samples extracted from vaccinated and challenged mice at 7 days (group 3) or 14 days post-challenge (groups 1 and 2), as well as viral yields in lung and nasal turbinates in the same groups. As shown in Fig. 4c, after the challenge mice vaccinated with mLNP-RBD or LNP-1-RBD formulations were able to prevent SARS-CoV-2 replication, significantly decreasing the number of genomic *RdRp* and subgenomic *N* SARS-CoV-2 RNA copy numbers compared to unprotected mice from PBS-treated control group. This observation correlated with the analysis of viral yields in lung homogenates and nasal turbinates (Fig. 4d), where protected mice did not show infectious virus in lung and nasal turbinates compared to unprotected mice from control group. These data demonstrated the efficacy of mLNP-RBD and LNP-1-RBD formulations to protect mice against SARS-CoV-2 infection.

**Fig. 5:**
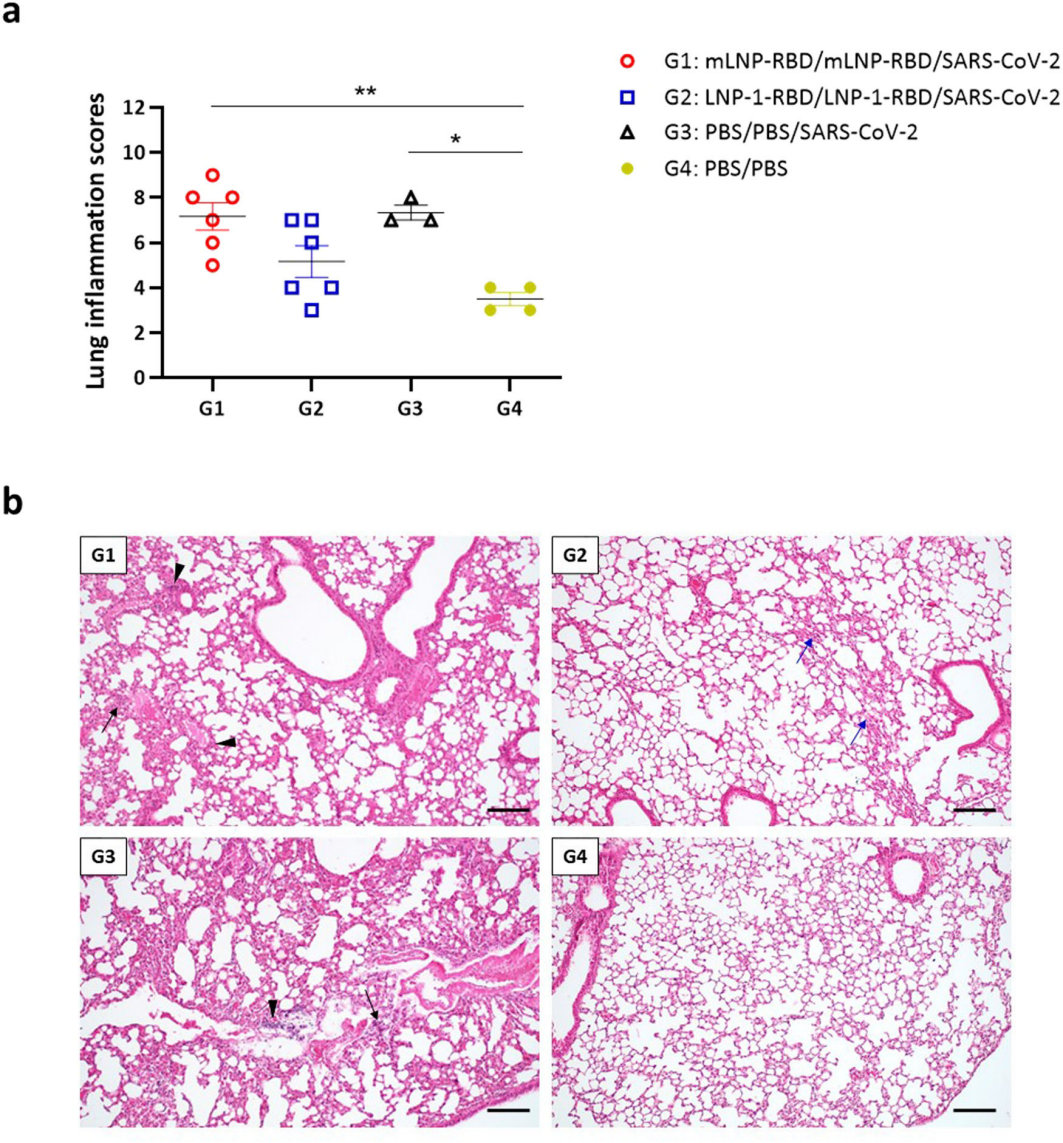
Lung pathology in vaccinated and challenged transgenic K18-hACE2 mice. **a** Lung inflammation scores observed in lung samples from vaccinated and challenged K18-hACE2 transgenic mice, and euthanized at day 7 (group 3) or day 14 post-challenge (groups 1, 2 and 4). Mean with SEM of cumulative histopathological lesion scores is indicated. An unpaired nonparametric Mann-Whitney test was performed.*p < 0.05; **p < 0.005. **b** Representative lung histopathological sections (H&E staining) from K18-hACE2 transgenic mice included in each of the experimental groups (scale bar: 200 µm). The severity and extent of inflammatory lung lesions observed in the immunized and challenged mice included in group 1 were similar to those described in the mice included in the non-immunized and challenged control group (group 3). Lesions included the presence of mild to moderate diffuse thickening of the alveolar septa, occasional small multifocal alveolar mononuclear cell infiltrates (black arrows) or mild multifocal perivascular and peribronchiolar mononuclear infiltrates (black arrowheads). Mice included in group 2 showed the lowest inflammatory scores. These animals showed only some lung areas with mild thickening of the alveolar septa (blue arrows) together with occasional small focal perivascular mononuclear infiltrates, showing an appearance highly similar to that observed in the PBS-treated non-challenged mice (group 4).

Finally, we also analyzed the histopathological lesions observed in the lungs of immunized mice after virus challenge. As shown in Fig. 5a, the lung inflammation scores observed in the mice of the different groups that were challenged were higher than those obtained in non-challenged PBS-treated mice, indicating some lung damage induced by SARS-CoV-2 infection. Among them, mice belonging to group 2 (LNP-1-RBD) displayed the lowest scores. Challenged animals exhibited a range of mild to moderate inflammatory lesions including thickening of the alveolar septa, alveolar mononuclear cell infiltrates or perivascular and peribronchiolar mononuclear infiltrates (Fig. 5b).

### mLNP-RBD and LNP-1-RBD formulations differentially regulate the proinflammatory cytokine and chemokine profiles in lung from vaccinated and challenged transgenic K18-hACE2 mice

**Fig. 6:**
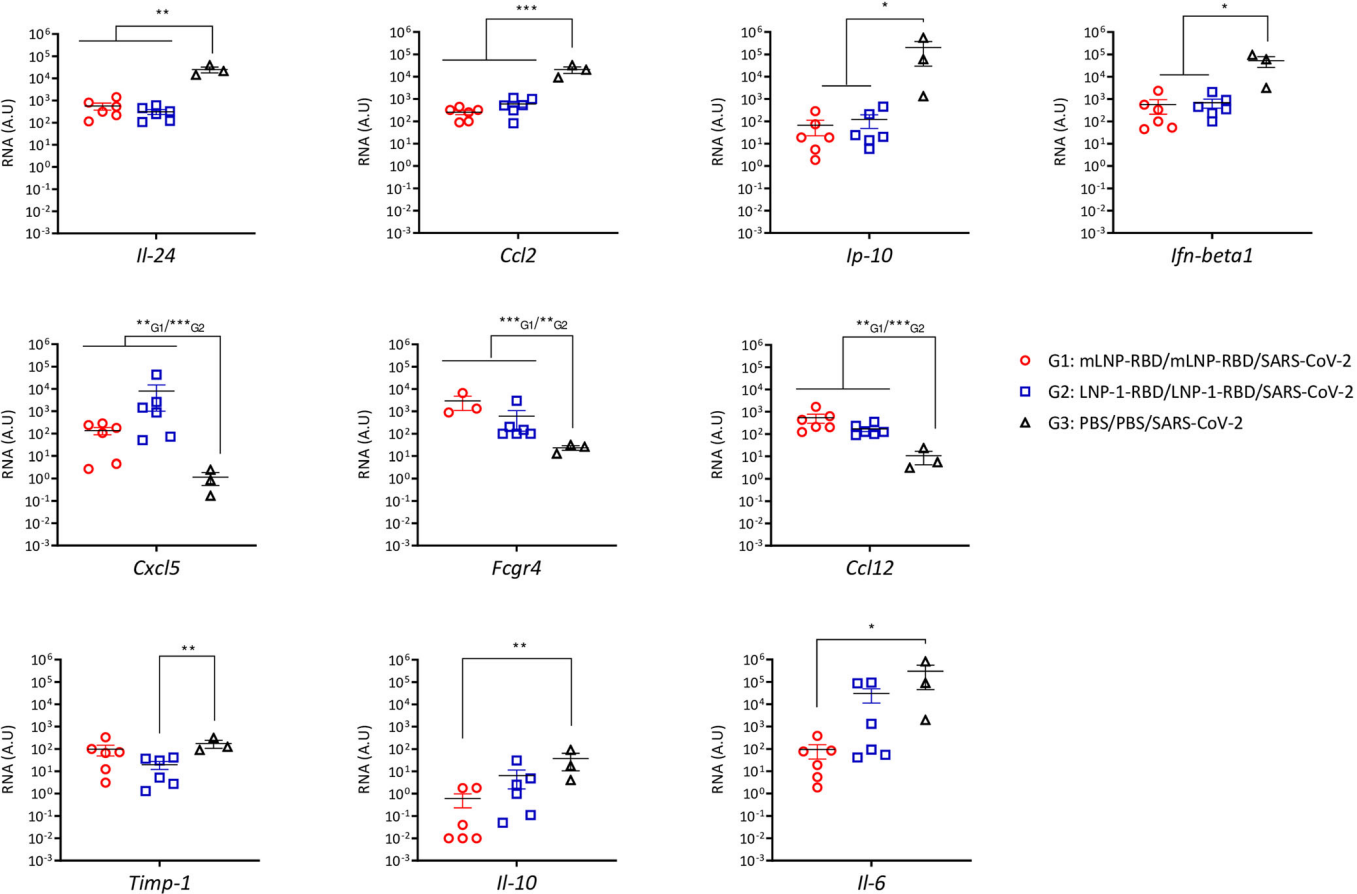
mLNP-RBD and LNP-1-RBD formulations differentially regulate proinflammatory cytokine and chemokine profiles in lung from vaccinated and challenged transgenic K18-hACE2 mice. Proinflammatory cytokines and chemokines were detected by RT-qPCR in lungs from individual mice at 14 days (groups 1 and 2) or 7 days (group 3) after SARS-CoV-2 challenge. Mean RNA levels (in A.U.) with SEM from duplicates of each lung sample is represented; relative values are referred to uninfected mice (group 4). An ordinary one-way ANOVA of transformed data followed by Tukey’s multiple comparison test was performed. *p < 0.05; **p < 0.005; ***p < 0.001.

Since an extensive upregulation of different proinflammatory cytokines has been correlated with COVID-19 disease progression and severity^11–13^, we evaluated the effect of both mRNA-RBD LNP formulations on the chemokine and proinflammatory cytokine expression profiles induced after SARS-CoV-2 challenge. For this, mRNA levels of key cytokines in lung homogenates from vaccinated mice were analyzed by RT-qPCR at 7 (group 3) or 14 days post-challenge (groups 1 and 2) (Fig. 6 and Supplementary Fig. 2). Overall, we observed a differential regulation of the proinflammatory cytokine and chemokine expression levels by both nanocarriers. Compared with the lungs of infected K18-hACE2 control mice, we observed a significant reduction of *Il-24*, *Ccl2*, *Ip-10* and *Ifn-beta1* RNA levels in both vaccinated groups, whereas *Cxcl5*, *Fcgr4* and *Ccl12* RNA levels were significantly increased in mLNP-RBD and LNP-1-RBD groups (Fig. 6). In addition, we observed a reduction of *Timp-1* in the group vaccinated with LNP-1-RBD and of *Il-10* and *Il-6* in the group vaccinated with mLNP-RBD (Fig. 6). No differences compared to control group were observed in the RNA expression levels of *Tnf-α, Ifn-ɣ, Il-12beta* and *Ifit27* (Supplementary Fig. 2).

## Discussion

The FDA and EMA approval of two mRNA vaccines, mRNA-1273 and BNT162b2, for the control of COVID-19 pandemic has opened a new era for vaccine development. As of 26 November 2023, a total of 13.59 billion vaccine doses have been administered globally and almost 90% of them were based on the mRNA technology (https://covid19.who). These vaccines have shown great safety and immunogenicity profiles and continue to exhibit high protection efficacy against severe disease, hospitalizations and death across age groups and in diverse populations^14^.

mRNA vaccines have brought about a great revolution in the vaccine field owing to a number of advantages compared with other platforms including: (i) their simplicity and flexibility in antigen design; (ii) strong target specificity; (iii) potential to elicit both cell-mediated and humoral immune responses; (iv) rapid, scalable and low-cost manufacturing practices allowing the production of different mRNAs with minimal adaptations and safety due to the almost zero probability of random genome integration; and (v) the transient expression of the encoded antigens^15–17^. All these features make the mRNA technology an ideal platform for developing vaccines against emerging viral infections with high efficacy. However, broad application of mRNA is still restricted by the need of improved delivery systems.

Currently, more than 50% of the mRNA-based vaccines in development use LNP formulations as delivery system. Nevertheless, the intellectual property (IP) landscape regarding LNP formulations is complex, and the supply chain of some of the components is restricted^18–20^. Innovations in the design of alternative delivery vehicles with higher antigen-delivery efficiency, improved stability and potency are highly desirable for mRNA vaccines.

In this study we have evaluated the immunogenicity and efficacy of the mRNA-RBD, a vaccine candidate against MAD6 SARS-CoV-2 virus encoding the full receptor binding domain (RBD: aas 330-532) of the SARS-CoV-2 S protein when delivered by different nanocarriers in mice. All the NE- and NC-based candidates failed to induce RBD-specific IgG binding and SARS-CoV-2-specific neutralizing antibodies after homologous prime/boost administration in C57BL/6 mice. In contrast, both LNP-based mRNA-RBD formulations induced SARS-CoV-2-specific binding and neutralizing antibodies at levels similar to those obtained in mice similarly injected with BNT162b2 vaccine. In transgenic K18-hACE2 mice, homologous prime/boost administration of mLNP-RBD or LNP-1-RBD formulations fully protected mice from morbidity and mortality against SARS-CoV-2 infection. The protection observed correlated with: (i) the induction of high levels of S- and RBD-specific IgG binding antibodies and of neutralizing antibodies against SARS-CoV-2 MAD6 and VoCs before challenge; (ii) a decrease in the number of genomic *RdRp* and subgenomic *N* SARS-CoV-2 RNA copy numbers; (iii) a reduced infection in lung and nasal turbinates; and (iv) a down-regulation of some chemokines and proinflammatory cytokines. We detected a significant reduction of *Il-24*, *Ccl2*, *Ip-10* and *Ifn-beta1* RNA levels in both LNP-vaccinated groups compared to challenged K18-hACE2 control mice. These proteins have key roles in JAK/STAT, NFKB and TGFB pathways and were targets for SARS-CoV-2 miRNAs^21^. In addition, we observed a significant reduction of *Timp-1* RNA levels in the group vaccinated with LNP-1-RBD. TIMP-1 is a secreted protein that blocks metalloproteinases which has been reported to be involved in lung inflammation^22^. Related to the severe lung inflammation and deficient function described in SARS-CoV-2-infected humanized ACE2-transgenic mice^23^, we also detected a significant down-regulation of *Il-10* and *Il-6* RNA levels in mLNP-RBD group. In contrast, we detected the up-regulation of the *Ccl12*, *Cxcl5* and *Fcgr4* RNA levels in LNP-vaccinated groups compared to challenged control mice. Increases in the infiltration of immune cells, along with high levels of CCL12^24^ and CXCL5, a chemokine responsible for neutrophil recruitment^25^, have been reported in SARS-CoV-2-infected mice as a hallmark of the pulmonary antiviral innate immune response. Moreover, there is evidence pointing towards the potential role of CXCL5 as a protective cytokine in SARS-CoV-2 infection^26^. In a different model of respiratory infection, CXCL5 has been reported to affect B lymphocyte accumulation in the lungs of influenza virus-infected mice by regulating the expression of the CXCL13 chemokine and orchestrating the antiviral innate and adaptive immune responses^27^. The *Fcgr4* gene has been reported to confer protection to lethal influenza virus infection^28^.

The histopathological analysis reveals the presence of some lesions in the lungs of vaccinated mice, indicating that LNP-based mRNA-RBD vaccine candidates are not sterilizing and, consequently, the SARS-CoV-2 virus was able to infect and replicate to some extent in the lungs of immunized mice. However, immunizations were able to fully protect the animals.

The use of trimeric mRNAs-RBD for vaccination is also an advantage over monomers or dimers, as the natural occurring S protein exists in the virus particle as a trimer. We took advantage of the bacteriophage T4 fibritin located at the C-terminus of RBD (330–532 aa) for trimerization of the RBD protein. Significantly, the intracellular processing of the RBD protein varied according to the formulation. The mLNP-RBD contributes to a more rapid release of the RBD protein from the treated cells than LNP-1-RBD, with an impact in the immune response triggering higher levels of binding antibodies. The importance of this type of mRNA-RBD design and formulations is critical since in C57BL/6 mice some formulations are quite effective in triggering specific immune responses and others are ineffective. This might be related to the stability, uptake and release of the mRNAs by the cell, to the composition and/or size of the different formulations or to the specific sensing of each formulated mRNA-RBD vaccine candidate. In this regard, the in vivo effectiveness gradient from high to low is the following: LNP > NE = NC, highlighting the importance of the design of the nanocarriers developed, and the need of formulation improvement and development of alternative LNP-derived nanoparticles. These results are in consonance with previously reported findings suggesting that the cationic or ionizable nature of the nanoparticles can lead to preferential cellular or humoral immune responses, respectively. This is mainly attributed to the structural and chemical characteristics of the nanoparticles, highly influenced by their components^29^. Cationic NEs (and, in consequence, NCs) result in the absence of humoral responses. Authors attribute this behavior to the enhanced lysosomal escape in DCs and the higher biodistribution in secondary lymphoid organs, which induced cellular-preferred immunity. On the other hand, LNPs are known to circumvent lysosome degradation favoring humoral-biased immune responses. The role of the LNP carrier as a potent adjuvant, and the innate and adaptive immune cell dynamics after administration of mRNA-LNP vaccines have also been recently reported^30^. The size and surface properties of the LNPs used for mRNA delivery can impact the biodistribution, lymphatic transport and cellular uptake by innate immune cells. Activated monocytes, macrophages and DCs are among the most relevant cell types involved in mRNA-LNP uptake, synthesis of the encoded protein and antigen presentation in lymphoid tissues to drive the adaptive immune response^30^. Moreover, studies aimed to optimize LNPs for vaccination purposes suggest the high influence of the pKa of the ionizable lipid or the PEGylation^31,32^.

The incorporation of modified nucleosides in mRNA vaccines is a crucial design element that impacts stability, translation efficiency and the nature of the immune response contributing to the success of mRNA vaccines. However, the precise contributions of the nucleoside-modified mRNA vs. LNP components to the overall immune response have not yet been clearly ascertained. In a rhesus macaque model, local inflammation occurs at the site of injection of either nucleoside-modified or unmodified mRNA-LNP vaccines, with infiltration of several immune cell types such as neutrophils, DCs and monocytes^33,34^. In our study both unmodified mLNP-RBD and LNP-1-RBD vaccine candidates fully protect transgenic mice against infection, indicating that both the intrinsic immunostimulatory nature of unmodified RBD-mRNA and the composition of the LNP used to deliver the mRNA vaccine could synergistically induce productive immunity.

Since we used a trimeric soluble form of RBD as immunogen, the preferential effect of the administration of the different formulated mRNA-RBD would be the induction of humoral immune responses as shown in this investigation. Thus, the analysis of the SARS-CoV-2-specific T cell response has not been performed, although the induction of innate and Th1-biased cellular immunity can also play a role in the efficacy of the mRNA vaccines against SARS-CoV-2. On the other hand, from the evidence provided by the neutralization assays, a good correlation between levels of NAbs and protection against SARS-CoV-2 MAD6 isolate is observed. The fact that protected animals elicited NAbs levels against the Delta variant comparable to those obtained against the MAD6 isolate suggests that the protective immunity induced by our formulated mRNA-RBD approach could be extended to different VoCs, as previously reported^35^. Indeed, the levels of NAbs against the Omicron, BQ1.1 and XBB1.5 variants fall within the range of 10^3^–10^5^.

All these observations established a strong capacity of mRNA-RBD formulated in LNPs as highly effective vaccine candidates to control SARS-CoV-2 infection, morbidity and mortality.

## Methods

### mRNA production and manufacturing

For this study we designed three different mRNA immunogens termed RBDepi-mRNA, RBD-mRNA and RBD-mRNA* (Supplementary Fig. 1A) that were synthetized by Prof. Thielemans’s Lab (Vrije Universiteit Brussel, Brussels, Belgium). The RBDepi-mRNA contains the highly immunogenic motif from the RBD (aas: 439–505) inserted into the post-fusion trimeric core of the S protein. The RBD-mRNA encodes the RBD domain of SARS-CoV-2 S protein (aa: 330–532) modified by the inclusion of a foldon trimerization domain derived from T4 fibritin to force trimerization and enhance its immunogenicity^36–39^. RBD-mRNA lifetime and translatability into the encoding protein was enhanced by in vitro transcription of the RBD-mRNA using modified N1-Methyl-Pseudouridine (RBD-mRNA*). In all mRNA constructs the 5′ and 3′ untranslated regions (UTRs) are derived from beta globin proteins^40–44^. 3D models built for RBDepi- and RBD-encoded proteins were shown to be stable up to 120 ns molecular dynamics simulation performed with GROMACS^45^ in standard conditions, during which the RBD immunogenic loops preserved their original conformations. Synthetic gene fragments for the three designed constructs were ordered (gBlocks^TM^, IDT, Coralville, IA, USA) and cloned into the pLMCT plasmid for in vitro transcription of GLP-grade mRNA. The RBD DNA sequence was codon-optimized and linked to a signal peptide (SP) from DC-LAMP at the 5′-terminus. As mRNA delivery carriers, six prototypes for the encapsulation of the SARS-CoV-2 RBD mRNA were selected after the screening of more than 300 formulation candidates. For this, a Target Product Profile (TPP) was implemented, considering that the selected prototypes should have (i) a particle size below or around 200 nm; (ii) low polydispersity index; (iii) high mRNA association efficiency; (iv) long-term stability; and (v) alignment with regulatory requirements.

### Preparation of mRNA-loaded nanosystems

1,2-dioleoyl-3-trimethylammonium propane chloride salt (DOTAP), 1,2-dioleoyl-sn-glycero-3-phosphoethanolamine (DOPE) and 1,2-distearoyl-sn-glycero-3-phosphocholine (DSPC) were purchased from Avanti Polar Lipids (Alabaster, AL, USA). C12-200 (HCl salt) and (R)-methoxy-polyethyleneglycol-2000-carbamoyl-di-O-myristyl-sn-glyceride (DMG-PEG2000) were a generous gift from Alnylam Pharmaceuticals (Cambridge, MA, USA). D-Lin-MC3-DMA (MC3) was purchased from TargetMol Chemicals (Wellesley Hills, MA, USA). Plant-derived cholesterol (SyntheChol^TM^ USP/NF. Ph.Eur., JP) and dextran sulfate (DX, sodium salt) were purchased from Sigma-Aldrich SAFC (St. Louis, MO, USA). Captex8000NF (tricaprylic acid) was purchased from ABITEC Corporation (Columbus, OH, USA). d, l-α-tocopherol (Vitamin E) was obtained from BASF (Mannheim, Germany). Tween 80 was acquired from Merck Millipore (Burlington, MA, USA). PEG_5_-b-PGA_10_ Na (poly(ethyleneglycol)-block-poly l-glutamic acid sodium salt; PEG-PGA) was obtained from Polypeptide Therapeutic Solutions (Valencia, Spain).

All nanoparticles were formulated by solvent-displacement technique, consisting of the controlled mixing of an ethanol phase (containing an appropriate amount of lipid components) with an aqueous phase (containing the mRNA)^46^. NEs were prepared by mixing both aqueous and organic phases using magnetic stirring. Briefly, an organic phase was prepared by dissolving different lipids (including DOTAP, DOPE, Vitamin E, Captex8000NF or Tween 80) in ethanol. The resulting ethanol solution was added over an aqueous phase (RNase-free water), under magnetic stirring (1400 rpm), and further incubated under stirring for 5 minutes. Then, mRNA solution was added over the previously formed blank NE solution, under magnetic stirring at 700 rpm for 10 s, at a nitrogen-to-phosphate (N/P) ratio of 4:1. The NCs were produced by adding a polymeric solution over the preformed NE-mRNA carrier, under magnetic stirring at 700 rpm for 10 s, at a v/v ratio of 1:5 (polymer to mRNA). In the case of the LNPs, aqueous solution (mRNA in citrate buffer, pH 4) and organic phase (containing an appropriate amount of the ionizable lipid, phospholipid, cholesterol, surfactant or PEGylated lipid) were simultaneously injected in a microfluidic system (NanoAssemblr® Benchtop; Precision NanoSystems, Vancouver, Canada) at a 3:1 aqueous to organic flow rate ratio and 9 mL/min total flow rate.

Resulting formulations were concentrated using an Amicon® Ultra 0.5 mL Centrifugal Filters Ultracel® -100K (Merck Millipore), following manufacturer’s recommendations. The formulations were characterized by size, polydispersity and zeta-potential (Zetasizer Nano ZS; Malvern Instruments, Malvern, UK). Final mRNA concentration and RNA encapsulation efficiency were estimated using Quant-iT RiboGreen RNA assay kit (Invitrogen, Waltham, MA, USA), following manufacturer’s instructions, and agarose gel assay.

### Cells and viruses

The highly transfectable 293T cells (ATCC 293T-CRL-3216), Vero-E6 (ATCC C1008; Vero 76, clone E6) and VeroE6/TMPRSS2 cells, which constitutively express the serin protease TMPRSS2 under geneticin selection (kindly provided by Prof. Enjuanes and Dr. Honrubia, CNB-CSIC, Madrid, Spain), were grown in Dulbecco’s Modified Eagle’s medium (DMEM) supplemented with 0.1 mM non-essential amino acids (Sigma-Aldrich), 2 mM l-glutamine (Merck, Kenilworth, NJ, USA), 100 U/mL penicillin/100 µg/mL streptomycin (Sigma-Aldrich) and 10% heat-inactivated fetal calf serum (FCS; Sigma-Aldrich) and maintained in a humidified air 5% CO_2_ atmosphere at 37 °C. VeroE6/TMPRSS2 cells, a cell line highly susceptible to SARS-CoV-2 infection and used to produce large viral stocks, were also supplemented with 1 mg/mL geneticin (G418, Sigma-Aldrich). Immature monocyte-derived dendritic cells (MDDCs) were obtained from peripheral blood mononuclear cells (PBMCs) from healthy donors as previously reported^47^.

SARS-CoV-2 strain MAD6 (provided by Prof. Enjuanes and Dr. Honrubia, CNB-CSIC, Madrid, Spain) is a virus harvested from a nasopharyngeal swab from a 69-year-old male COVID-19 patient from Hospital 12 de Octubre in Madrid and was prepared as previously reported^48^. SARS-CoV-2 MAD6 isolate is similar to the B.1 strain but includes the D614G mutation in the S protein and has been previously reported^49^. The SARS-CoV-2 Alpha (B.1.1.7) variant (strain hCoV-19/France/IDF-IPP11324i/2020, 20I/501Y.V1) was supplied by the National Reference Centre for Respiratory Viruses hosted by Institut Pasteur (Paris, France) and headed by Pr. Sylvie van der Werf. The human sample from which the virus was isolated has been provided by Dr. Foissaud, Hôpital d’Instruction des Armées Percy (HIA Percy; Clamart, France. The SARS-CoV-2 Beta (B.1.351) variant (hCoV-19/France/PDL-IPP01065i/2021, 10H/501Y.V2) was supplied by the National Reference Centre for Respiratory Viruses hosted by Institut Pasteur (Paris, France) headed by Pr. Sylvie van der Werf, and the human sample from which the virus was isolated has been provided by Dr. Besson from the Bioliance, st-Herblain Laboratory (Saint-Herblain, France). Moreover, VoCs B.1.1.7 and B.1.351 were supplied through the European Virus Archive Global (EVAG) platform, a project that has received funding from the European Union’s Horizon 2020 research and innovation programme under grant agreement No 653316, and were kindly provided through Dr. Juan García-Arriaza (CNB-CSIC, Madrid, Spain). The SARS-CoV-2 Delta (B.1.617) variant (SARS-CoV-2, Human, 2021, Germany ex India, 20A/452R) was supplied by Dr. Andreas Nitsche from the Robert Koch Institute (German Federal Institute for Infectious and Non-Communicable Diseases, Berlin, Germany) through the European Virus Archive Global (EVAG) platform, and was kindly provided through Dr. Juan García-Arriaza (CNB-CSIC, Madrid, Spain). The SARS-CoV-2 B.1.1.529 Omicron BA.1 variant (hCoV-19/Belgium/rega-20174/2021, EPI_ISL_6794907) was supplied by Prof. Piet Maes from KU Leuven (Belgium) through Dr. Robbert Boudewijns and Dr. Kai Dallmeier (KU Leuven, Belgium) and provided to us through Dr. Juan García-Arriaza (CNB-CSIC, Madrid, Spain). SARS-CoV-2 omicron BQ.1.1 (EPI_ISL_15653663) and XBB.1.5 (EPI_ISL_16939528) variants were kindly provided by Prof. Rafael Delgado (Hospital Universitario 12 de Octubre, Madrid, Spain) through Dr. Juan García-Arriaza (CNB-CSIC, Madrid, Spain).

SARS-CoV-2 viral stocks were grown on VeroE6/TMPRSS2 cells^50^ by two passages and were mycoplasma-free (PlasmoTest, InvivoGen, San Diego, CA, USA). Deep sequencing on a MiSeq platform (Illumina, San Diego, CA, USA) confirmed that virus stocks included no other adventitious agents. Viral titers were determined by standard plaque assay and by median tissue culture infectious dose assay by the method of Spearman–Kärber (TCID50) in Vero-E6 cells^51^. The experiments with these viruses were conducted in the BSL-3 facilities of the CNB-CSIC (Madrid, Spain), according to institutional guidelines.

### Expression of SARS-CoV-2 RBD protein from naked and formulated mRNAs

Time-course expression of RBD protein was assayed by flow cytometry and western-blotting analyses. For this, 293T cells seeded on 6-well plates (1 × 10^6^ cells/well) were transfected with 5 µg of each naked mRNA (RBDepi-mRNA, RBD-mRNA and RBD-mRNA*) using Lipofectamine-2000 (Invitrogen) according to manufacturer’s recommendations. At 3, 6 and 24 h post-transfection, 2.5 × 10^5^ cells were harvested for western-blotting analysis and the remaining cells were collected and processed for flow cytometry analysis.

For flow cytometry analysis, cells were filtered through a cell strainer using PBS 1× (Ca^-^/Mg^-^), washed once, resuspended in flow cytometry staining buffer [FACS buffer: PBS 1×-1% bovine serum albumin (BSA)-2 mM EDTA] and seeded in a 96-well plate. After centrifugation (1500 rpm, 5 min) and supernatant removal, cells were incubated with the live/dead fixable red dye (1:200; Invitrogen) at 4 °C in the dark for 30 min, washed twice with FACS buffer and fixed/permeabilized with BD Cytofix/Cytoperm (BD Biosciences, San Jose, CA, USA) at 4 °C for 20 min. After, cells were centrifuged (1500 rpm, 5 min), washed twice with PermWash (PW) 1× buffer (diluted in FACS buffer; BD Biosciences) and blocked with PBS 1×-3% BSA at 4 °C for 30 min. Then, cells were incubated with a rabbit polyclonal anti-SARS-CoV-2 spike/RBD antibody (5 µg/mL; Sino Biological, Beijing, China) at 4 °C for 30 min in the dark. Next, cells were washed twice with PW 1× and incubated with a secondary anti-rabbit IgG (H + L)-PE antibody (1:100; Beckman Coulter, Brea, CA, USA). After incubation for 30 min in the dark at 4 °C, cells were washed twice with PW 1×, resuspended in FACS buffer and acquired in a FC500 1 Laser flow cytometer (Beckman Coulter). The analysis of the data was performed using FlowJo software (Version 10.4.2; Tree Star, Ashland, OR, USA). RBD score was calculated using the geometric Mean Fluorescence Intensity (gMFI) values within the “live cells” gate applying the formula: No. RBD^+^ cells × gMFI/No. live cells. For western-blotting analysis, cellular pellets and supernatants from 2.5 × 10^5^ 293T-transfected cells were obtained as previously described^52^, fractionated by 8% Sodium Dodecyl Sulfate-Polyacrylamide Gel Electrophoresis (SDS-PAGE) and analyzed using a rabbit polyclonal anti-SARS-CoV-2 spike/RBD antibody (1:2000; Sino Biological), followed by goat anti-rabbit-horseradish peroxidase (HRP) (1:5000; Sigma-Aldrich) to evaluate RBD expression. The immunocomplexes were detected by enhanced-chemiluminescence (ECL Plus; GE Healthcare, Chicago, IL, USA).

The efficiency of delivery and RBD expression from the different nanocarriers containing the RBD-mRNA were assayed in monolayers of 293T cells grown in 24-well plates and transfected with 5 µg of the different mRNA formulations. Cellular pellets and supernatants were obtained at 6 h post-transfection and RBD expression was analyzed by western-blotting using a rabbit polyclonal anti-SARS-CoV-2 spike/RBD antibody. All blots were processed in parallel and derive from the same experiments.

The delivery and expression of RBD-mRNA from the different nanocarriers was also analyzed at 6 and 24 h after transfection of human monocyte-derived dendritic cells (hMDDCs) by flow cytometry as previously described^53^. Briefly, hMDDCs obtained from blood monocytes were harvested from 96-well plates (10 × 10^5^ cells per well) and transfected with formulated RBD-mRNA nanocarriers at concentrations ranging from 3 to 10 ug per well. After 3 h, cells were washed twice and fresh medium containing maturation cocktail (IL-6, IL-1β, PGE2 and TNFα) was added. At 6 and 24 h post-transfection, cells were collected and RBD expression was analyzed by flow cytometry following a protocol similar to that described for 293T-transfected cells with some minor variations: (i) FcR blocking reagent (Miltenyi Biotec, Bergisch Gladbach, Germany) was used instead of BSA for intracellular blocking; (ii) an anti-rabbit IgG (H + L) antibody stained with Alexa fluor 647 (Invitrogen) was used as secondary antibody; (iii) cells were acquired in a FACS Canto II cytometer (BD Biosciences, San Jose, CA, USA); and (iv) cell cytotoxicity was also evaluated using the LIVE/DEAD™ Fixable Near IR Reagent (Invitrogen).

### Ethics statement

The immunogenicity and efficacy mouse studies were approved by the Division of Animal Protection of the Comunidad de Madrid (Spain; PROEX 161.5/20 and 169.4/20) and by the Ethical Committee of Animal Experimentation (CEEA) of the CNB-CSIC. Animal procedures were in accordance with international guidelines and with Spanish law under the Royal Decree RD 53/2013.

Blood was harvested from volunteer blood donors at the Banc de Sang i Teixits (BST) (Barcelona, Spain) after signing a written informed consent. This study obtained the approval of the Committee of Ethics and Clinical Investigation of the Hospital Clinic Universitari, Barcelona, Spain (HCB/2020/0387).

### Immunogenicity study in C57BL/6 mice

Female C57BL/6OlaHsd mice (6–8 week-old) purchased from Envigo Laboratories (Sant Feliu de Codines, Barcelona, Spain) and housed in the animal facility of CNB-CSIC (Madrid, Spain) were used to evaluate the immunogenicity of the different nanocarriers. For this, six groups of animals (*n* = 5) received two doses of 40 µg of the RBD-mRNA formulated in NE-1 (G1), NE-2 (G2), NC-1 (G3), NC-2 (G4), mLNP (G5) or LNP-1 (G6) by intramuscular (i.m.) route at days 0 and 21. Mice primed and boosted with mRNA-LUC formulated in LNP-1 were used as control group (G7). Serum samples from mice vaccinated with two doses of 5 μg of BNT162b2 human vaccine (mRNA vaccine from Pfizer-BioNTech, kindly provided by Dr. Montserrat Plana, University of Barcelona, Spain) at days 0 and 21 by i.m. route were used as benchmark data. Blood was harvested by submandibular bleeding at 20 days post-prime (d20) and 21 days post-boost (d42) for the analysis of anti-RBD IgG binding and SARS-CoV-2 neutralizing antibodies.

### Efficacy study in K18-hACE2 transgenic mice

Female transgenic K18-hACE2 mice (9 week-old), expressing the human angiotensin converting enzyme-2 (ACE2) receptor, were purchased from Jackson Laboratory (Bar Harbor, ME USA; 034860-B6.Cg-Tg(K18-ACE2)2Prlmn/J, genetic background C57BL/6J × SJL/J)F2) and used to determine the efficacy of selected nanocarriers against SARS-CoV-2 infection. For this, two groups of animals (*n* = 6) received two doses of 40 µg of the RBD-mRNA formulated in mLNP (G1) or LNP-1 (G2) by i.m. route at days 0 and 21. Mice primed and boosted with PBS were used as control groups (G3 and G4). Serum samples from mice vaccinated with two doses of 5 μg of BNT162b2 vaccine at days 0 and 21 by i.m. route were used as benchmark data. Blood was harvested by submandibular bleeding at 20 days post-prime (d20) and 21 days post-boost (d42) for the analysis of anti-S and anti-RBD IgG binding and SARS-CoV-2 neutralizing antibodies. At day 47, animals from G1 to G3 were challenged by intranasal (i.n.) route with a lethal dose (1 × 10^5^ PFU) of SARS-CoV-2 (MAD6 strain) under isoflurane anesthesia. Animals from G4 remained unchallenged. Mice were monitored daily for weight, general health and survival for 14 days and those with more than a 25% of weight loss were euthanized by cervical dislocation and different organs harvested and processed (see below). At the end of the study animals that survived were sacrificed and lungs, nasal turbinates and serum samples were harvested as previously described^54^. All experiments were performed under a laminar flow cabinet in the biosafety level 3 (BSL-3) facilities at the Centro de Investigación en Sanidad Animal (CISA)-Instituto Nacional de Investigación y Tecnología Agraria y Alimentaria (INIA-CSIC) (Valdeolmos, Madrid, Spain).

### Enzyme-linked immunosorbent assay

Individual serum samples from C57BL/6 and transgenic K18-hACE2 mice were evaluated for the presence of anti-SARS-CoV-2 S and RBD proteins binding IgG antibodies by Enzyme-Linked Immunosorbent Assay (ELISA) as previously reported^55^. Briefly, individual sera were 3-fold serially diluted in duplicates and incubated with 2 μg/mL of recombinant SARS-CoV-2 S or RBD purified proteins (kindly provided by Dr. Casasnovas, CNB-CSIC, Madrid, Spain). The S and RBD proteins derived from the Wuhan-Hu-1 strain (GenBank accession number MN908947.3). Total binding IgG titers were determined as the last serum dilution that gives 3 times the mean optical density value measured at 450 nm (OD_450_ value) of the control group (endpoint titer).

### SARS-CoV-2 neutralization

Live-virus SARS-CoV-2 neutralizing antibodies in individual serum samples from C57BL/6 and transgenic K18-hACE2 mice were determined using a microneutralization test (MNT) assay in a BSL-3 laboratory as previously described^56^. Briefly, individual sera were 2-fold serially diluted in duplicates in DMEM-2% fetal bovine serum (FBS; Gibco, Waltham, MA, USA) and incubated at a 1:1 ratio with 100 TCID50 of SARS-CoV-2 MAD6 isolate or SARS-CoV-2 VoCs Alpha (B.1.1.7), Beta (B.1.351), Delta (B.1.617.2), Omicron (B.1.1.529), BQ1.1 and XXB1.5 in 96-well tissue culture plates for 1 h at 37 °C. Next, mixtures of serum samples and SARS-CoV-2 virus were added in duplicates to Vero-E6 cells seeded in 96-well plates (30,000 cells/well), and plates were incubated for 3 days at 37 °C in a 5% CO_2_ incubator. After, cells were fixed for 1 h with 10% formaldehyde (Sigma-Aldrich) and stained with crystal violet (Sigma-Aldrich). After drying the plates, crystal violet was diluted in H_2_O-10% SDS and optical density at 570 nm was measured in a luminometer. To obtain the neutralization titers, half maximal inhibitory concentration (IC_50_) and 95% confidence intervals (95% CI) were calculated using a nonlinear regression model fit with settings for log agonist versus normalized response curve using GraphPad Prism v9.4.1 Software (GraphPad Software, San Diego, CA, USA).

### Analysis of SARS-CoV-2 and cytokine/chemokine RNAs by reverse transcription-quantitative polymerase chain reaction (RT-qPCR)

Lungs from transgenic K18-hACE2 mice were homogenized using a gentleMACS dissociator (Miltenyi Biotec, Bergisch Gladbach, Germany) in 2 mL of RLT buffer (Qiagen, Hilden, Germany) plus β-mercaptoethanol (Sigma-Aldrich). Total RNA was extracted from 0.6 mL of homogenized lung tissue using the RNeasy Mini kit (Qiagen), following the manufacturer’s instructions. Total SARS-CoV-2 genomic (*RdRp*) and subgenomic (*N*) RNA copy numbers (copies/µl) were quantified by RT-qPCR as previously reported^57^ and using the SARS-CoV-2 One-Step RT-PCR Kit II, RdRp and N genes, IVD (NZYTech, Lisboa, Portugal), following the manufacturer´s recommendations (Supplementary Table 1). The mRNA expression levels of different cytokine/chemokine genes [*Il-24, Ccl2, Ip-10 (CXCL10), Ifn-beta1, Cxcl5, Fcgr4, Ccl12, Timp-1, Il-10*, *Il-6, Tnf-α, Ifn-ɣ, Il-12beta and Ifit27*] were analyzed using specific Taqman probes (Thermo Fisher Scientific, Waltham, MA, USA; Supplementary Table 1). Data were acquired in a 7500 real-time PCR system (Applied Biosystems, Waltham, MA, USA) and analyzed using the 7500 software v2.0.6. Relative RNA arbitrary units (A.U.) were determined relative to a negative control group (PBS-uninfected mice) using the 2−ΔΔCt method and cellular 28 S rRNA was used for normalization. Samples were tested in duplicate.

### Analysis of SARS-CoV-2 virus yields by plaque assay

SARS-CoV-2 infectious virus in lungs and nasal turbinates from transgenic K18-hACE2 mice at 7 or 14 days post-challenge was analyzed by standard plaque assay, as previously described^58,59^. Briefly, collected lungs were weighed and homogenized with a gentleMACS dissociator (Miltenyi Biotec) in 2 mL of PBS buffer. Nasal turbinates were obtained after nasal washes with 0.1 mL of PBS. Next, undiluted and 10-fold serial dilutions of either homogenized lung tissues or nasal turbinates were added in triplicates to VeroE6 cells seeded in 12-well plates (5 × 10^5^ cells/well). After 1 h of virus adsorption, the inoculum was withdrawn and plates were incubated at 37 °C, 5% CO_2_ in 2:1 DMEM 2×-4% FBS:Avicel^®^ RC-591 (carboxymethylcellulose sodium and microcrystalline cellulose, DuPont Nutrition Biosciences ApS, Koebenhavn, Denmark). After 4 days of incubation, cells were fixed with 10% formaldehyde (Sigma-Aldrich) for 1 h, the supernatant was withdrawn, and viral plaques were visualized by the addition of 0.5% crystal violet (Sigma-Aldrich). SARS-CoV-2 titers were defined as PFUs/gram of lung tissue or PFUs/mL of nasal turbinates.

### Lung histopathology

The left lung lobe of K18-hACE2 transgenic mice was excised, fixed by immersion in 10% zinc formalin solution (Sigma-Aldrich) for 48 h and then paraffin-embedded. Lung Section (4 µm) were stained with haematoxylin and eosin (H&E) and then microscopically evaluated for lung damage by a single veterinary pathologist (Veterinary Pathology Department, CISA-INIA) who did not know the identity of the mice. To determine the presence and severity of histopathological lesions, scoring parameters of lung inflammation based on previous studies of SARS-CoV-2 infection in mouse models were used^60^. The histopathological parameters evaluated were the following: alveolar haemorrhages; alveolar oedema; perivascular oedema; inflammatory cell infiltration in alveoli; alveolar septal thickening (interstitial pneumonia); peribronchial/peribronchiolar and perivascular mononuclear infiltrates; bronchi/bronchioles with epithelial necrosis, detached epithelium or inflammatory cells in the lumen (bronchitis/bronchiolitis); syncytia formation and pleural thickening or cytopathic effect in pneumocytes. The histopathological parameters were graded according to a semi-quantitative scoring system as follows: 0: no lesion; 1: minimal lesion; 2: mild lesion; 3: moderate lesion; and 4: severe lesion. The total lung inflammation score of each animal was the result of the cumulative histopathological lesion scores observed in each animal. Individual scores were used to calculate the average of each group.

### Statistical procedures

Statistical evaluations were performed with GraphPad Prism v9.4.1 Software (GraphPad Software). Flow cytometry data from transfected hMDDCs were analyzed using nonparametric (Mann–Whitney or Wilcoxon signed rank test) or parametric (Student’s *t*-test) tests as appropriate. An unpaired nonparametric Mann-Whitney test of transformed data was used for statistical analysis of binding IgG antibody titers, and an ordinary one-way ANOVA of transformed data followed by Tukey’s multiple comparison test was performed for the live virus NT_50_ neutralizing titers and for the analysis of mRNA levels and SARS-CoV-2 virus yields. An unpaired nonparametric Mann-Whitney test was employed for the statistical evaluation of lung histopathological scores. Statistical significance is indicated as follows: *p < 0.05; **p < 0.005; ***p < 0.001. For the in vivo assays, we calculated the sample size for a one-way ANOVA to detect an effect size of 0.9 with a confidence level of 95% and a power of 95%. This effect size corresponds to a change of a single group of 2.8 times the standard deviation. This results in 5 individuals per group, which is the sample size used in the immunogenicity assay in C57BL/6 mice. Foreseeing an increase in the standard deviation in the efficacy assay in hACE2Tg mice due to its more complicated execution, we increased the group sample size of the efficacy experiment to 6.

### Supplementary information

Supplementary Material

## Data Availability

The raw data supporting the conclusions of this work will be available upon request, without undue reservation.
