# Author Correction: Modulating the immune response to SARS-CoV-2 by different nanocarriers delivering an mRNA expressing trimeric RBD of the spike protein: COVARNA Consortium

**DOI:** 10.1038/s41541-024-00882-4

**Published:** 2024-05-18

**Authors:** Laura Marcos-Villar, Beatriz Perdiguero, Shubaash Anthiya, Mireya L. Borrajo, Gustavo Lou, Lorenzo Franceschini, Ignasi Esteban, Pedro J. Sánchez-Cordón, Carmen Zamora, Carlos Óscar S. Sorzano, Luis Jordá, Laia Codó, Josep L. Gelpí, Marta Sisteré-Oró, Andreas Meyerhans, Kris Thielemans, Francisco Martínez-Jiménez, Núria López-Bigas, Felipe García, María J. Alonso, Montserrat Plana, Mariano Esteban, Carmen Elena Gómez

**Affiliations:** 1 Department of Molecular and Cellular Biology, Centro Nacional de Biotecnología, Consejo Superior de Investigaciones Científicas, Madrid, Spain; 2 Centro de Investigación Biomédica en Red de Enfermedades Infecciosas (CIBERINFEC), Instituto de Salud Carlos III (ISCIII), Madrid, Spain; 3 Center for Research in Molecular Medicine and Chronic Diseases (CiMUS), Campus Vida, Universidade de Santiago de Compostela, Santiago de Compostela, Spain; 4 Laboratory for Molecular and Cellular Therapy, Department of Biomedical Sciences, Vrije Universiteit Brussel, Brussels, Belgium; 5 AIDS Research Group, Institut d’Investigacions Biomèdiques August Pi i Sunyer (IDIBAPS), Hospital Clinic, University of Barcelona, Barcelona, Spain; 6 Veterinary Pathology Department, Centro de Investigación en Sanidad Animal, Instituto Nacional de Investigación y Tecnología Agraria y Alimentaria, Consejo Superior de Investigaciones Científicas, Madrid, Spain; 7 Biocomputing Unit and Computational Genomics, Centro Nacional de Biotecnología, Consejo Superior de Investigaciones Científicas, Madrid, Spain; 8 Barcelona Supercomputing Center (BSC), Barcelona, Spain; 9 Department of Biochemistry and Molecular Biomedicine, University of Barcelona, Barcelona, Spain; 10 Infection Biology Laboratory, Department of Medicine and Life Sciences, University Pompeu Fabra, Barcelona, Spain; 11 Institució Catalana de Recerca i Estudis Avançats (ICREA), Barcelona, Spain; 12 Institute for Research in Biomedicine (IRB Barcelona), The Barcelona Institute of Science and Technology, Barcelona, Spain; 13 Centro de Investigación Biomédica en Red en Cáncer (CIBERONC), Instituto de Salud Carlos III, Madrid, Spain; 14 Infectious Diseases Department, Hospital Clínic, University of Barcelona, Barcelona, Spain

**Keywords:** Viral infection, RNA vaccines

Correction to: *npj Vaccines* (2024) **9**:53; 10.1038/s41541-024-00838-8, published online 06 March 2024

In this article, the author name Núria López-Bigas was incorrectly written as Nuria López-Vigas. The original article has been corrected.

