# Supplementary material for: Modulating the immune response to SARS-CoV-2 by different nanocarriers delivering an mRNA expressing trimeric RBD of the spike protein: COVARNA Consortium
Source: NPJ Vaccines. 2024 Mar 6;9:53. doi: 10.1038/s41541-024-00838-8 (PMC10918104; doi:10.1038/s41541-024-00838-8)
Supplement: Supplementary file 1 — Supplementary Material [file 41541_2024_838_MOESM1_ESM.pdf]

Supplementary Figure 1

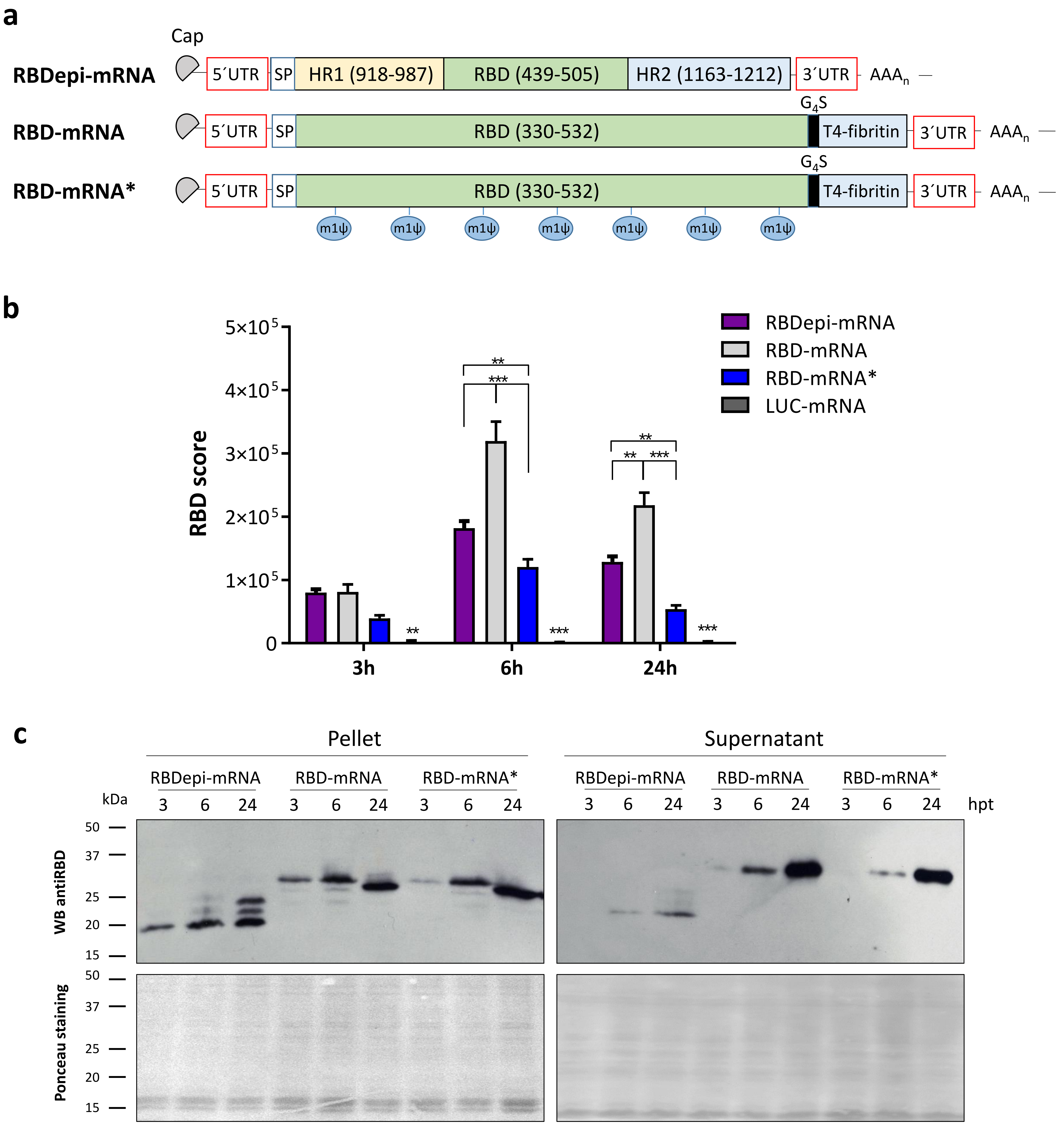

Supplementary Figure 1. Time-course expression of SARS-CoV-2 RBD protein after transfection of 293T cells with different naked mRNAs. (A) Scheme of the structure of the three different mRNAs expressing the full receptor binding domain (RBD: aas 330-532) of the SARS-CoV-2 spike protein (RBD-mRNA and RBD-mRNA*) or its highly immunogenic motif (RBDepi-mRNA). **(B)** Detection of RBD expression in transfected 293T cells at 3, 6 and 24 h post-transfection (h.p.t.) by flow cytometry using a rabbit polyclonal anti-SARS-CoV-2 spike/RBD antibody (5 µg/mL). Geometric Mean Fluorescence Intensity (gMFI) values on the "live cells" gate were used to calculate the RBD score by applying the formula: No. RBD^+ cells × gMFI/No. live cells. Cells transfected with naked LUC-mRNA were used as negative control. Mean with standard error of the mean (SEM) is represented. A multiple *t* test was performed. ** $p < 0.005$; *** $p < 0.001$. **(C)** Kinetics of RBD expression in cellular pellets and supernatants from transfected 293T cells by western-blotting using a rabbit polyclonal anti-SARS-CoV-2 spike/RBD antibody (upper panels). Ponceau staining (lower panels) was used as loading control. All blots derive from the same experiment and were processed in parallel.

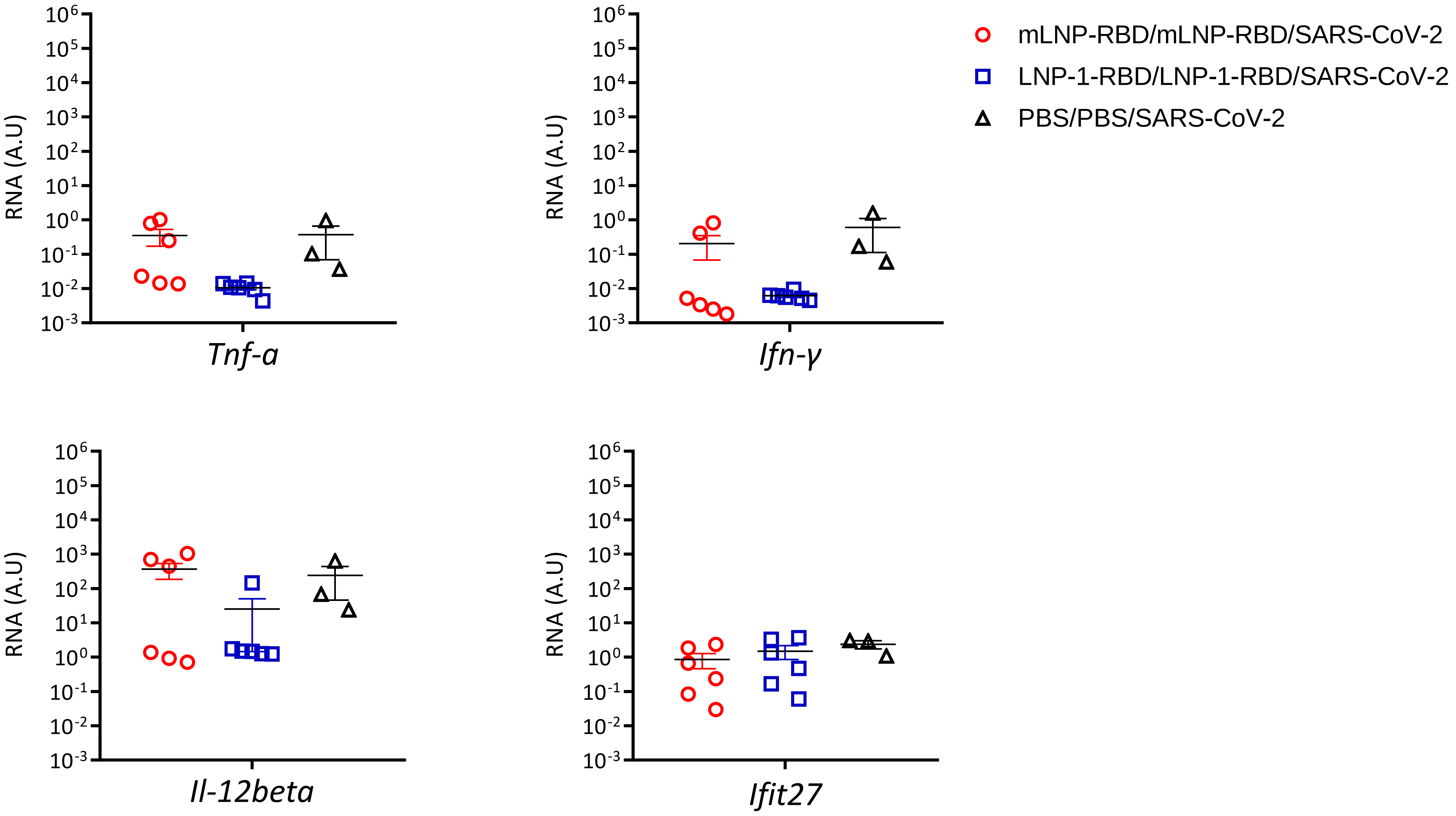

Supplementary Figure 2. mLNP-RBD and LNP-1-RBD formulations do not change significantly *Tnf-α, Ifn-γ, Il-12beta* and *Ifit27* gene expression profiles in lungs from vaccinated and challenged transgenic K18-hACE2 mice. Proinflammatory cytokines and chemokines were detected by RT-qPCR in lungs from individual mice at 14 days (groups 1 and 2) or 7 days (group 3) after SARS-CoV-2 challenge. Mean RNA levels (in A.U.) with standard error of the mean (SEM) from duplicates of each lung sample is represented; relative values are referred to uninfected mice (group 4). An ordinary one-way ANOVA of transformed data followed by Tukey's multiple comparison test was performed. ns > 0.05**.**

Supplementary Table 1

Target	Taqman probes
Il-24	Mm00474102_m1
Ccl2	Mm00441242_m1
Ip-10 (Cxcl10)	Mm00445235_m1
Ifn-beta1	Mm00439552_g1
Cxcl5	Mm00436451_g1
Fcgr4	Mm00519988_m1
Ccl12	Mm01617100_m1
Timp-1	Mm01341361_m1
Il-10	Mm00439616_m1
Il-6	Mm00446190_m1
Ifn-γ	Mm01168134_m1
Tnf-α	Mm00443258_m1
Il-12beta	Mm01288989_m1
Ifit27	Mm00508912_m1
	**Primers/probe sets**
28s	PROBE[JOE] 5´ TAGTAGCTGGTTCCCTCCGAAGTTTCCCT 3´<br>Rev 5´ CGAGAGCAGCTATCCT 3´<br>Fwd 5´GGCGAAAGACTAATCGAACCAT 3´
	**Kit**
RdRp	NZYTech SARS-CoV-2 One-Step RT-PCR Kit II,
N	RdRp and N genes, IVD (MD04871)

Supplementary Table 1. Taqman probes used for the detection of proinflammatory cytokines and chemokines. Primers/probe sets used for the detection of 28S (housekeeping gene). Detection kit used for the quantification of the total SARS-CoV-2 genomic (*RdRp*) and subgenomic (*N*) RNA copy numbers.

Uncropped blots of Figure 1b

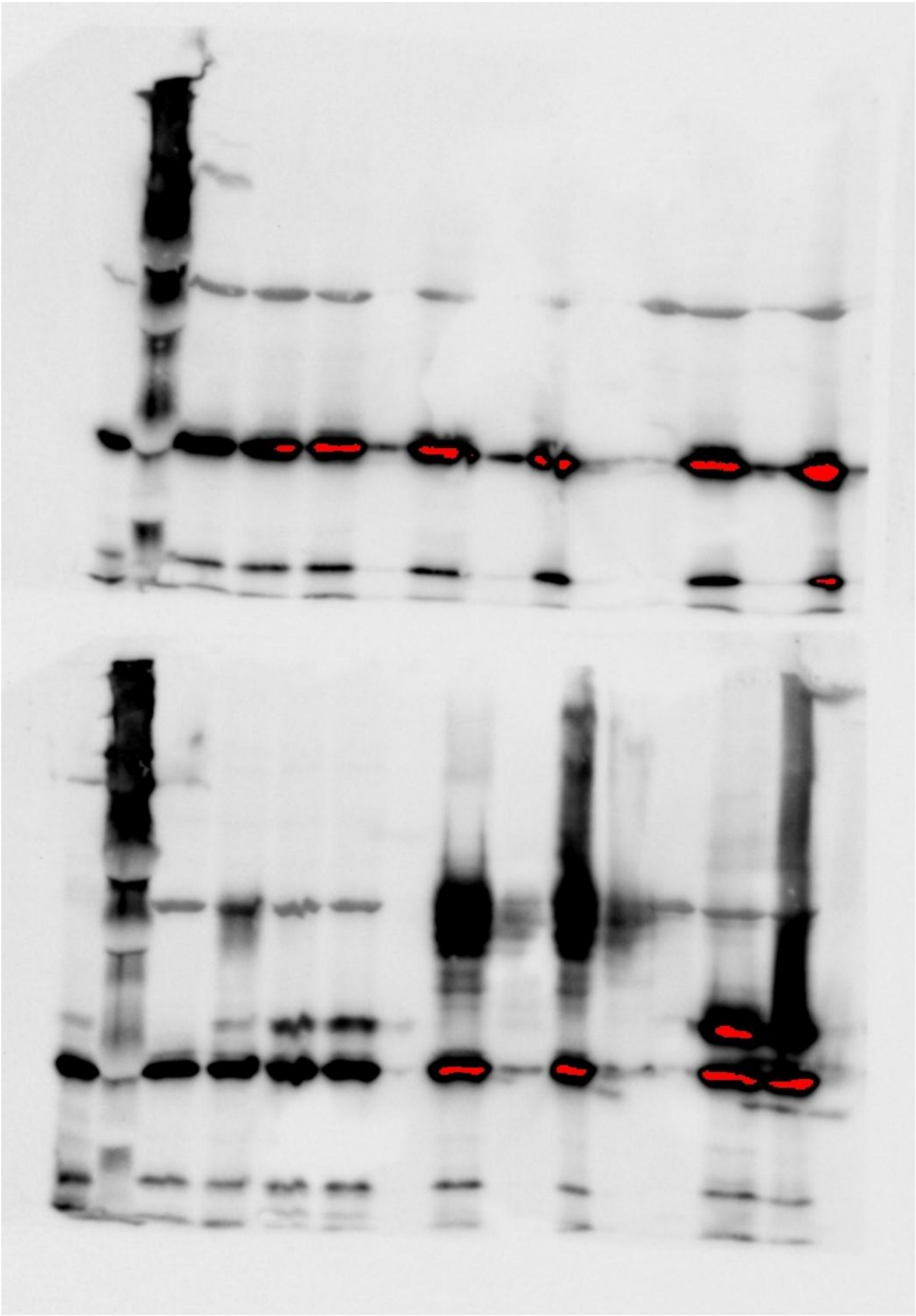

RBD staining

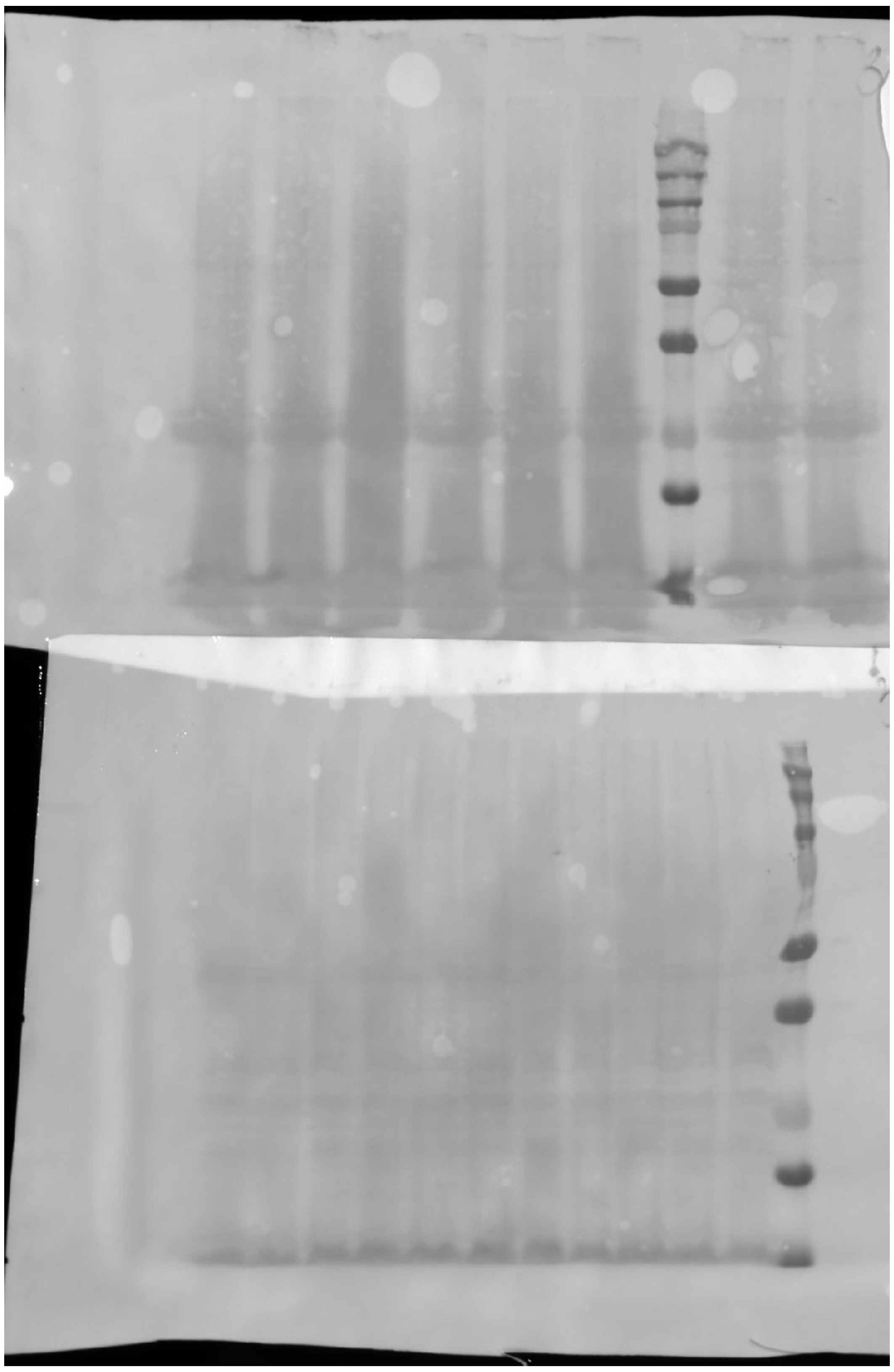

Ponceau staining

Uncropped blots of Supplementary Figure 1c

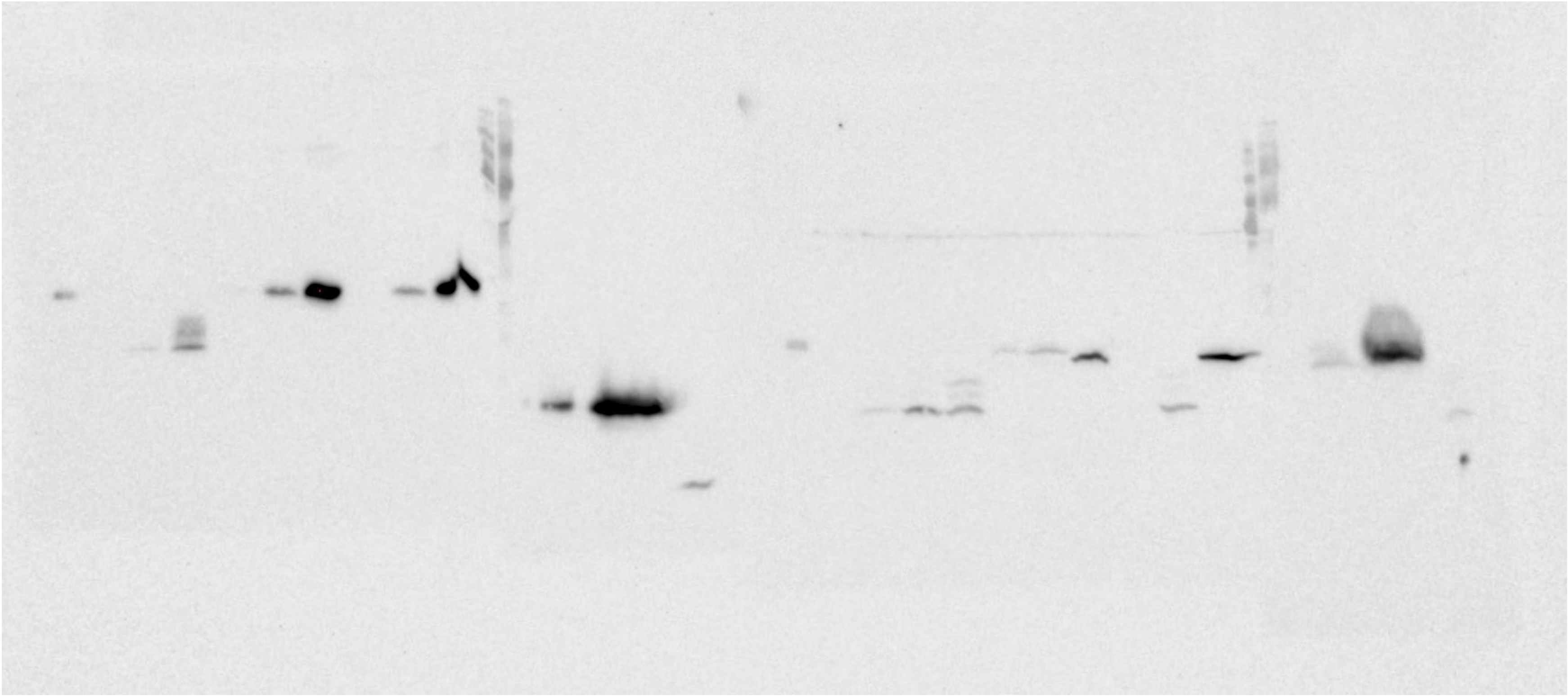

RBD staining

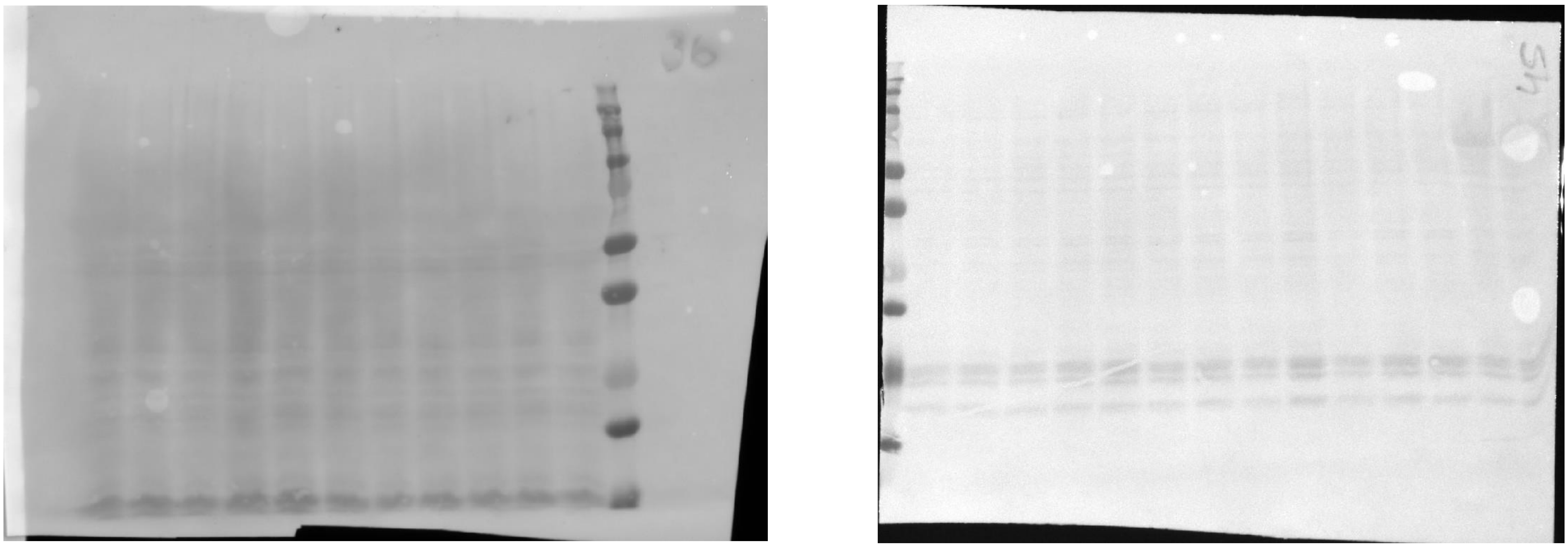

Ponceau staining
